# Recent Advances in Metal–Organic Frameworks for Gas Sensors: Design Strategies and Sensing Applications

**DOI:** 10.3390/s26030956

**Published:** 2026-02-02

**Authors:** Aviraj M. Teli, Sagar M. Mane, Sonali A. Beknalkar, Rajneesh Kumar Mishra, Wookhee Jeon, Jae Cheol Shin

**Affiliations:** 1 Division of Electronics and Electrical Engineering, Dongguk University-Seoul, Seoul 04620, Republic of Korea; avteli.teli@gmail.com (A.M.T.); sonaliabeknalkar@gmail.com (S.A.B.); 2 Department of Fiber System Engineering, Yeungnam University, 280 Dehak-Ro, Gyeongsan 38541, Republic of Korea; manesgar99@ynu.ac.kr; 3 Department of Physics, Yeungnam University, Gyeongsan 38541, Republic of Korea; 4 Department of Semiconductor, Convergence Engineering, Sungkyunkwan University, Suwon 16419, Republic of Korea; wookie92@skku.edu

**Keywords:** metal-organic frameworks, fundamentals of MOFs, properties, gas sensors, selectivity

## Abstract

Gas sensors are essential in areas such as environmental monitoring, industrial safety, and healthcare, where the accurate detection of hazardous and volatile gases is crucial for ensuring safety and well-being. Metal–organic frameworks (MOFs), which are crystalline porous materials composed of metal nodes and organic linkers, have recently emerged as a versatile platform for gas sensing due to their adjustable porosity, high surface area, and diverse chemical functionality. This review provides a detailed overview of MOF-based gas sensors, beginning with the fundamental sensing mechanisms of physisorption, chemisorption, and charge transfer interactions with gas molecules. We explore design strategies, including functionalization and the use of composites, which improve sensitivity, selectivity, response speed, and durability. Particular attention is given to the influence of MOF morphology, pore size engineering, and framework flexibility on adsorption behavior. Recent developments are showcased across various applications, including the detection of volatile organic compounds (VOCs), greenhouse gases, toxic industrial chemicals, and biomedical markers. Finally, we address practical challenges such as humidity interference, scalability, and integration into portable platforms, while outlining future opportunities for real-world deployment of MOF-based sensors in environmental, industrial, and medical fields. This review highlights the potential of MOFs to transform next-generation gas sensing technology by integrating foundational material design with real-world applications.

## 1. Introduction

Monitoring and measuring gases in diverse environments, such as industrial areas, cities, hospitals, and homes, has become increasingly important [1]. Although gases are often invisible and odorless, they can significantly affect human health, ecosystems, and industrial processes [2]. Imbalances in specific gases can have serious effects. Toxic gases such as carbon monoxide, hydrogen sulfide, and ammonia pose health risks even at low levels, causing respiratory and neurological issues [3]. Greenhouse gases notably contribute to global warming, while nitrogen oxides and sulfur dioxide can lead to acid rain and smog, threatening biodiversity and water supplies [4]. Attention is needed for industrial gases, which may cause explosions or corrosion but also improve process and product quality [5]. Advances in gas sensing technologies are crucial for ensuring safety, regulatory compliance, and operational efficiency across these sectors. Pollution sensors help track pollutants contributing to climate change and atmospheric damage [6]. In industry, gas sensors are key for process control, leak detection, and safety, especially in petrochemical, mining, and semiconductor industries [7]. They are also used in healthcare for non-invasive diagnostics and measuring key gases like oxygen and anesthetics. Despite their widespread use, traditional gas sensors, such as metal–oxide semiconductors, electrochemical, catalytic, and optical sensors, often face significant limitations [8]. These include high operating temperatures, limited selectivity, slow response and recovery times, sensitivity to humidity, and challenges related to long-term stability. However, monitoring gas levels is crucial not only for protecting the environment but also for healthcare diagnostics and ensuring industrial safety. Next-generation gas sensors employ metal–organic frameworks (MOFs), known for their extremely high surface areas and porosity, facilitating effective gas adsorption [9]. Their pore chemistry can be tailored for selective binding to specific analytes, with structural design and post-synthetic modifications allowing functional tunability. Under normal conditions, MOFs offer a flexible platform for developing sensitive sensors with rapid responses, low detection thresholds, high selectivity, and high sensitivity [10]. Their compatibility with various transducers further boosts their suitability for practical applications.

MOFs have attracted considerable attention since their discovery in the late 1990s, as they can be tuned in structure, possess exceptional physical and chemical properties, and exhibit a modular design [11,12]. Such structures are being studied in numerous applications, including gas storage, separation, catalysis, drug delivery, and, more importantly, gas and biological sensing [13]. MOFs have been utilized in sensing applications due to their large surface area, tunable microenvironment, chemical functionality, and the ability to alter pore size in response to an external stimulus [14]. MOFs are constructed from metal-containing secondary building units (SBUs) that serve as structural coordination centers. These may be individual metal ions, such as Zn^2+^, Cu^2+^, and Co^2+^, or they may be multinuclear metal–oxo clusters, like Zr_6_O_4_(OH)_4_ in UiO-66 [15]. The topography of the metal node plays a significant role in determining the overall topology of the framework [16]. Furthermore, metal identity can confer additional functionalities, such as redox activity, catalytic centers, or luminescence, which are particularly advantageous for sensing applications [17]. The use of these linkers and SBUs forms highly ordered crystalline materials with 1D, 2D, and 3D networks as the topology [18]. The modular design of frameworks allows almost unlimited flexibility in the choice of structure and functional characteristics of MOFs [19]. Brunauer–Emmett–Teller (BET) surface areas are reported to be very large, exceeding 10,000 m^2^/g [20]. This also has a large surface area, offering numerous adsorption sites that enable the generation of measurable responses with very small analytes. MOFs exhibit tunable pore structures through organic linkers or node design [21]. Interestingly, the apertures are ~3.4 Å and are highly selective for gases such as H_2_ and CO_2_, for which ZIF-8 is known to exhibit selectivity [22]. This selectivity enables MOFs to perform the functions of molecular sieves, providing size- and shape-selective sensing. Depending on the functional groups incorporated into the MOFs, the sites for chemical recognition are specific. Consider the case of UiO-66-NH_2_, which exhibits a high affinity for bonding with nitroaromatic compounds through hydrogen bonding and electron donor–acceptor interactions [23]. Thiol-MOFs exhibit high binding specificity to heavy metals, such as Hg^2+^, and can therefore be utilized in the environmental sensing of targeted elements [24]. The other cornerstone discovery in the chemistry of MOFs is the establishment of thermally and/or chemically stable structures, especially those of Zr and Al. MIL-101(Cr) and O-66 are porous in fundamental, acidic, and sticky conditions [25]. In-plane interlacing links and weakly tied out-of-plane links stacked in 2D MOFs. Cu-HHTP and many other 2D MOFs are conductive and can be adapted to chemiresistive sensing [26]. 3D MOFs have high surface areas due to highly interconnected pore networks and are ideal platforms for sensors. In rigid MOFs, such as UiO-66, the structure is retained even in the presence of a gas molecule, resulting in predictable sensing behavior. Plastic MOFs, also known as Breathing MOFs, are more flexible structures that swell or contract upon the binding of guest molecules, thereby increasing sensitivity as they move [27]. Luminescent MOFs (LMOFs) are produced through the incorporation of chromophoric linkers or lanthanide ions, and thus they have useful luminescent qualities [28]. Conductive MOFs feature redox-active linkers or nodes, producing p-conjugated structures or redox-active sites that confer electronic conductivity [29]. Catalytically Active MOFs may serve as enzyme mimics and identify particular targets due to a colorimetric variation during the catalysis process [30]. Their sensing potentials, such as optical, electrochemical, gravimetric, and catalytic/colorimetric detection, are built on the interactions between gas molecules in MOFs and their inherent flexibility. Fluorescence quenching of luminescent MOFs is capable of detecting nitroaromatics, explosives, or biomolecules [31]. Tb^3+^-based MOFs have also been studied in recent research and have been shown to exhibit significant luminescence shifts upon phosphate binding [32]. Another approach is an electrochemical sensing technique that utilizes conductive MOFs, where the transfer of charge can be observed when analytes are adsorbed onto the surface [33]. Gravimetric detection is used to measure mass changes when the analytes adsorb to MOF-coated quartz crystal microbalances (QCMs). ZIF-8-coated single-crystalline QCMs exhibit CO_2_ selectivity in comparison with N_2_ [34]. Furthermore, catalytic or colorimetric sensing applies MOFs that resemble peroxidase enzymes to give colorimetric measurements of H_2_O_2_ and glucose [35]. Although some reviews have already analyzed MOF-based gas sensors, such as in works by Soon Hyeong et al. [36], Feng et al. [37], Majhi et al. [38], and Yuan et al. [39], it is important to note that this review is highly comprehensive and application-oriented. It bridges the gap between molecular-scale MOF design principles and specific sensing principles and performance metrics in real-world settings. The review serves as a cross-disciplinary guide to the environmental, industrial, and biomedical applications of gas sensors, encompassing MOF-derived composites, hybrid heterostructures, flexible sensing platforms, and the ability to modify electronic structures. Additionally, we provide comparative illustrations and useful classifications of MOF-based sensors, offering a new perspective on evaluating their practicality in real-world applications. Advances in the concepts of stability and functionalization are bringing MOFs into practical use for environmental detection, biomedical diagnostics, and industrial safety. Together, these properties make MOFs versatile platforms for gas sensing, with adaptable structures that can be designed for specific detection tasks in environmental, industrial, and biomedical fields.

This review critically examines recent advances in gas sensing technologies, emphasizing metal–organic frameworks (MOFs). It outlines major material innovations, design approaches, and performance indicators that affect the sensitivity, selectivity, and stability of gas sensors.

## 2. Fundamentals of MOFs

Progress in stability and functionalization concepts is driving metal–organic frameworks (MOFs) closer to practical applications in various fields. The MOFs-Ni@MOFs-Fe was created using a two-step solvothermal process and then sulfurized to form MOFs-Ni@MOFs-Fe-S [40]. The resulting MOFs-Ni@MOFs-Fe-S consists of interdigitated, folded nanosheets with accessible active sites, thereby enhancing its electrochemical performance. These findings highlight the potential of MOFs-derived bimetallic sulfides to replace noble metals for cost-effective and efficient urea electrolysis. On the other hand, nickel–cobalt MOFs (NiCo-MOFs)/silver (Ag^+^) ion-doped films were synthesized directly on nickel foam (NF) via a solvothermal method, with the silver (Ag^+^) ion concentration varied [41]. Ag^+^ functions as a structure-directing agent, promoting the formation of smaller crystal domains and boosting redox activity by facilitating improved electron transport pathways. Interestingly, a new palladium-based MOFs (Pd-MOFs) was synthesized using Pd(NO_3_)_2_.2H_2_O and tetrakis(4-carboxyphenyl)porphyrin (TCPP) as a fluorescent nanoprobe for the ultrasensitive detection of the organophosphorus pesticide phorate [42]. The photoluminescent properties of the Pd-MOFs were explained by a unique bioluminescence-inspired mechanism, in which interaction with phorate increased the electron cloud density and the HOMO-LUMO gap, thereby stabilizing the system and enhancing fluorescence. Overall, the design and synthesis of MOFs with enhanced optoelectronic properties, achieved through the combined use of Pd and porphyrin-based ligands, has demonstrated a benchmark for MOFs-based sensors in environmental monitoring. Interestingly, the MOFs were grown on Ti_3_C_2_T_x_ MXene nanosheets to form a hybrid heterostructure, which enhances the conductivity and charge transport efficiency of MXenes [43]. To enhance humidity resistance without altering morphology or porosity, a surface ligand exchange reaction (SLER) using 5,6-dimethylbenzimidazole (DMBIM) was performed to impart hydrophobicity. Thus, MOFs/MXene hybrids with customized surface chemistry provide a robust foundation for gas sensing at trace levels, unaffected by humidity. Furthermore, MOFs are utilized as both a structural template and a chemical precursor for the synthesis of the Ni-Co alloy catalyst through controlled pyrolysis [44]. The MOFs with different Ni/Co ratios were preserved under hydrogen (H_2_) rather than a conventional inert atmosphere to produce well-dispersed bimetallic nanoparticles. The synergistic effect between Ni and Co regulates the electronic structure and promotes the catalytic activity of the nanomaterials. Significantly, the Co addition enhanced the thermal barrier and metal dispersion, thereby preventing the collapse of the structure of MOFs typically encountered during pyrolysis.

**Figure 1:**
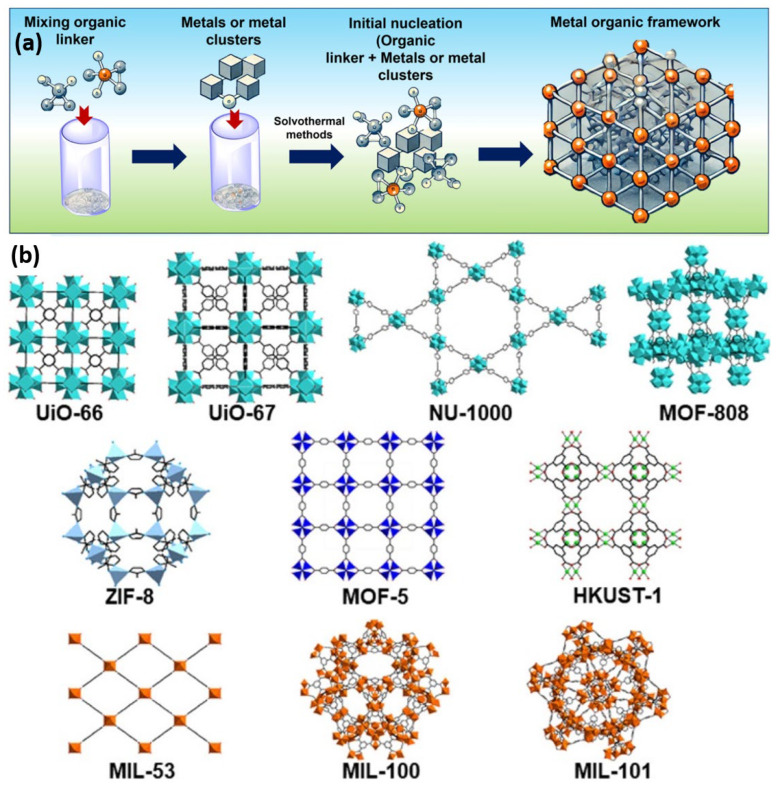
(**a**) Schematic of MOFs showing metal nodes coordinated with organic linkers, forming a porous and crystalline structure. Reproduced with permission from Ref. [45]. Copyright (2025) Elsevier. (**b**) Representative crystal structures of widely studied MOFs used in catalysis. Reproduced with permission from Ref. [46]. Copyright (2019) Elsevier.

Figure 1a provides a schematic illustration of a typical structure of MOFs, which is essential due to the coordination among metal nodes and organic ligands, constituting the extended crystalline networks of MOFs [45]. It illustrates that metal ions or metal clusters, such as Cu^2+^, Zn^2+^, and Fe^3+^, are bound together by multidentate organic ligands, including carboxylates, imidazolates, and phosphonates, to form a rigid and porous 3D structure. Long-range order and structural tunability, as illustrated in Figure 1a, are directly related to enhanced electron transport, ion diffusion, and catalytic activity, which are crucial parameters for the development of sensitive and selective electrochemical sensors. Furthermore, Figure 1a shows the modular nature of MOFs, which can be chemically tailored either through pre-synthesis ligand design or post-synthesis modification to add redox-active components, conductive frameworks, and specific binding groups. In the sensor development, the figure also graphically shows that MOFs can be used to immobilize electroactive species, such as nanoparticles and enzymes, thereby increasing the sensitivity, selectivity, and operational stability of the sensor systems. On the other hand, the crystal structures of the major MOFs that have been studied extensively for catalytic applications are presented in Figure 1b [46]. This visual comparison explores the structural diversity of MOFs, focusing on metal node composition, linker architecture, and pore geometries. The cases of UiO-66, UiO-67, NU-1000, MOF-808, MIL-101, ZIF-8, and HKUST-1 demonstrate that different metal–oxide cluster structures, such as Zr_6_O_8_, Cr_3_O, and Cu_2_, and organic linkers, can dramatically change the physical and chemical environment of potential catalytic sites. Figure 1b underlines the ability of MOFs to be strategically engineered to host catalytically active sites through open metal nodes, postsynthetic remodeling, or defect engineering. Interestingly, MOFs, such as UiO-66 and MOF-808, exhibit accessible Lewis or Bronsted acid sites at defect locations, whereas NU-1000 allows the incorporation of single metal centers or nanoclusters for redox and hydrogenation reactions. The shape-selective catalysis is directly proportional to the pore aperture, which is evident in the structures. Small-pore MOFs, such as ZIF-8, are well-suited for molecular sieving, while larger-pore MOFs, like MIL-101, support larger reactants, thereby increasing their range of reactions.

**Figure 2:**
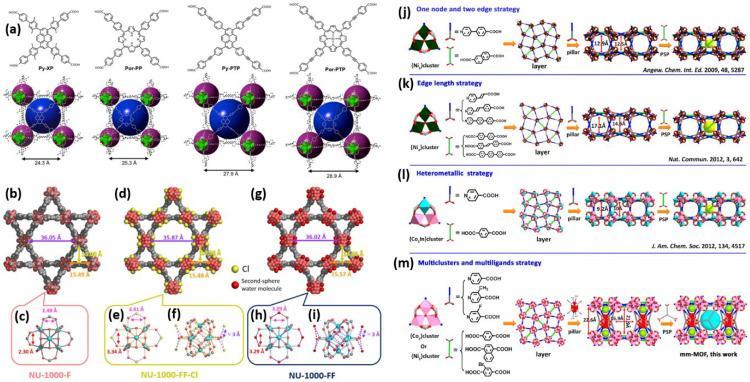
(**a**) Tetracarboxylate organic linkers (Py-XP, Por-PP, Py-PTP, and Por-PTP) and the associated crystal structures of the NU-110x series (NU-1101 to NU-1104), demonstrating systematic reticular expansion. There are big (blue) and small (purple) pores depicted, and the calculated pore diameters (dl, ds) increased through the series: NU-1101 (17.2, 9.5 Å), NU-1102 (20.5, 11.1 Å), NU-1103 (22.7, 12.7 Å), and NU-1104 (24.2, 13.5 Å). Reproduced with permission from Ref. [47]. Copyright (2015) Elsevier. (**b**–**i**) Structural evolution of the NU-1000 MOFs across three forms. (**b**,**d**,**g**) MOFs perspectives along the c-axis that depict mesopores and triangular micropores with respective Zr-Zr distances that reflect reversible pore contraction with the removal of ligands. (**c**) Zr_6_ node in NU-1000-F coordinated by ~3 formate ligands and one OH/OH_2_ pair, resulting in reduced accessibility and rigid structure of MOFs. (**e**,**f**) NU-1000-FF-Cl shows a node free of formate, coordinated by eight aqua ligands, with four nearby charge-balancing chloride ions that do not bind to Zr centers. (**h**,**i**) NU-1000-FF displays the ideal node configuration [Zr_6_(μ_3_-O)_4_(μ_3_-OH)_4_(H_2_O)_4_(OH)_4_] after base treatment, with maximal ligand lability and full node accessibility. Reproduced with permission from Ref. [49]. Copyright (2020) Elsevier. (**j**–**m**) Schematic representation of four strategies to form multicomponent MOFs using the integrated pillar-layered and pore-space partitioning (PL-PSP) methodology. (**j**) A classical MOFs design using a single type of node and two types of linkers. (**k**) Edge length strategy: modulation of pore sizes through variation in pillar and spacer ligand lengths. (**l**) Heterometallic strategy: incorporation of multiple metal ions to introduce structural and functional diversity. (**m**) Multiclusters/multiligands strategy: complex design using trinuclear and hexanuclear clusters of cobalt and three different ligands to form (3, 9, 12)-connected MOFs with hierarchical cage structures and superior C_2_H_2_/CO_2_ separation characteristics. Reproduced with permission from Ref. [50]. Copyright (2020) American Chemical Society.

In reticular chemistry, systematically extending linkers allows precise control over pore size and topology without compromising framework stability. The NU-110x series demonstrates this concept by gradually elongating linkers. Figure 2a illustrates the systematic reticular growth of the NU-110x series of zirconium-based MOFs, from NU-1101 to NU-1104, with increasingly longer tetracarboxylate linkers (Py-XP to Por-PTP) [47]. To illustrate the impact of linker modulation on topology retention, the NU-110x series maintains the ftw topology despite increased unit cell size and pore diameters. MOFs adopting the ftw topology are characterized by a binodal, edge-transitive network comprising 12-connected cuboctahedral nodes linked through 4-connected square or rectangular units [48]. The large (dl) and small (ds) pores expand to 24.2 Å and 13.5 Å, respectively, in NU-1101 and NU-1104, leading to higher geometric surface areas and pore volumes across the series. Notably, even with longer linkers, all MOFs retain non-interpenetrated cubic frameworks because the ftw topology is resistant to structural collapse. Figure 2b–i provides a visual comparison of the structural evolution of NU-1000 at three key stages, such as formate-containing (NU-1000-F), chloride-containing without formate (NU-1000-FF-Cl), and without both formate and chloride (NU-1000-FF) [49]. Figure 2b,d,g depicts the MOFs along the c-axis. The mesopore diameter, determined from Zr-Zr distances across the pores, decreases gradually from NU-1000-F to NU-1000-FF-Cl; however, a smaller reduction is observed in NU-1000-FF, indicating reversible pore contraction. This contraction is also captured in the triangular micropore edge and vertical dimensions. Most importantly, such structural dynamics are not caused by the collapse of the MOFs but by the reversible loss of water by aqua ligands when activated in the vacuum. The coordination pattern of NU-1000-F at Zr6, as depicted in Figure 2c, involves a Zr6 node coordinated by roughly 3 formate ligands and a single OH/OH_2_ pair. These formate ligands connect to the Zr centers, stabilizing the node and reducing ligand lability. NU-1000-FF-Cl is shown in Figure 2e,f with eight aqua ligands and four unbound chloride ions. The presence of bridging formate and terminal aqua ligands allows for dynamic ligand displacement, which is essential in catalytic reactions. The chlorides near the node are likely non-coordinating to Zr(IV) centers. In contrast, Figure 2h,i represents NU-1000-FF, where the chlorides are eliminated and four hydroxo ligands, which have been created through base treatment. This type of node organization is known to yield the optimal [Zr_6_(μ_3_-O)_4_(μ_3_-OH)_4_(H_2_O)_4_(OH)_4_]_8_^12+^ composition, which is theoretically the most Lewis-acidic and catalytically active MOFs. As shown in these structural comparisons, only NU-1000-FF-Cl and NU-1000-FF exhibit pore contraction upon solvent evacuation, a process facilitated by the removal of labile aqua ligands. Importantly, NU-1000-F does not experience this change because the formate bridging adds rigidity, resulting in a trade-off between passivating the nodes and maintaining framework flexibility. Furthermore, Figure 2j–m demonstrates the gradual development of strategies to create multicomponent MOFs, culminating in a novel combination of the pillared-layer (PL) strategy and pore-space partitioning (PSP) [50]. It begins with the traditional approach of a single node and two edges, in which the MOFs were constructed with minimal detail, as illustrated in Figure 2j. This was followed by the edge-length tuning method, which allowed control over pore sizes without altering topology, as depicted in Figure 2k. The heterometallic approach introduced multiple metals, increasing chemical diversity, as shown in Figure 2l. Interestingly, this innovation lies in combining multiclusters and multiligands via the PL-PSP method, enabling the construction of a robust (3,9,12)-connected llz topology of MOFs. This method effectively creates hierarchical cages and channels with different ligands and cobalt clusters. Interestingly, this signifies a paradigm shift toward multifunctional, modular MOFs and provides a model for designing next-generation structures with enhanced selectivity and tunability.

**Figure 3:**
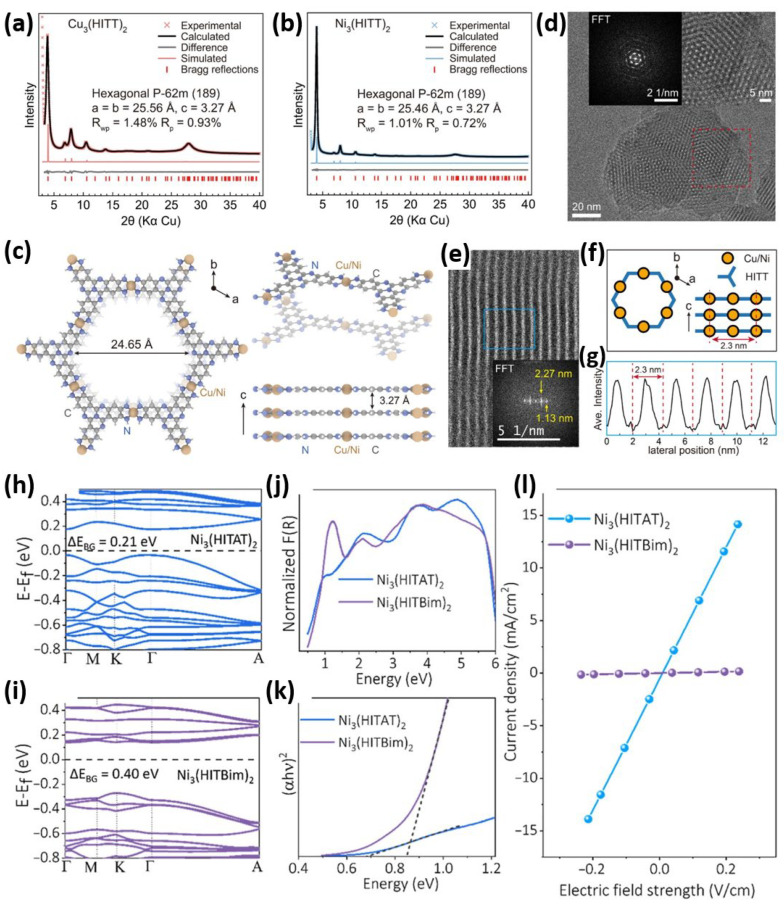
Experimental, simulated, and Pawley-refined powder X-ray diffraction (PXRD) patterns for (**a**) Cu_3_(HITT)_2_ and (**b**) Ni_3_(HITT)_2_, confirming high crystallinity and assignment to the hexagonal. (**c**) Simulated crystal structure (c-axis) and in the ab-plane (bottom) that show two-dimensional hexagonal sheets with eclipsed (AA) stacking. The carbon, nitrogen, and metal atoms (Cu/Ni) are depicted in gray, blue, and brown colors, respectively. Cryogenic high-resolution transmission electron microscopy (cryo-HRTEM) images of (**d**) Cu_3_(HITT)_2_ and (**e**) Ni_3_(HITT)_2_, in which well-ordered lattice fringes appear. Insets indicate Fast Fourier Transforms (FFT) corresponding to periodicity that is consistent with PXRD. (**f**) Schematic illustration of the three layers stacked along the c-axis with emphasis on the eclipsed stacking and p-p interactions. (**g**) Average intensity profile of the boxed area of the HRTEM image of Ni_3_(HITT)_2_, which indicates periodic contrast in line with the 2D lattice and stacking between the layers (2.3 nm). Reproduced with permission from Ref. [51]. Copyright (2024) American Chemical Society. (**h**,**i**) Charge transport properties of Ni_3_(HITAT)_2_ and Ni_3_(HITBim)_2_ MOFs. (**h**,**i**) Calculated electronic band structures of bulk Ni_3_(HITAT)_2_ and Ni_3_(HITBim)_2_, respectively, illustrating in-plane band dispersion for Ni_3_(HITAT)_2_ and suppressed dispersion for Ni_3_(HITBim)_2_, indicating stronger charge delocalization. (**j**) Diffuse reflectance spectra of both MOFs transformed using the Kubelka–Munk function to assess optical absorption. (**k**) Corresponding Tauc plots derived from the spectra, estimating optical band gaps of 0.68 eV for Ni_3_(HITAT)_2_ and 0.85 eV for Ni_3_(HITBim)_2_. (**l**) Room-temperature current density vs. electric field plots for pressed pellet samples of the two MOFs measured using a two-point probe configuration, indicating a significantly higher electrical conductivity in Ni_3_(HITAT)_2_ (44 mScm^−1^) compared to 0.5 mScm^−1^ for Ni_3_(HITBim)_2_. Reproduced with permission from Ref. [52]. Copyright (2023) American Chemical Society.

Figure 3a–g offers the two-dimensional conductive metal–organic frameworks (2D cMOFs) Ni_3_(HITT)_2_ and Cu_3_(HITT)_2_, emphasizing their crystallographic features, stacking patterns, and nanoscale morphology [51]. The PXRD experimental, simulated, and Pawley-refined for Cu_3_(HITT)_2_ and Ni_3_(HITT)_2_ patterns are presented in Figure 3a,b. Both experimental and refined data confirm the formation of highly crystalline, well-ordered 2D layered structures. Figure 3c,f depicts both the modeled crystal structure and a schematic of the eclipsed AA stacking in each MOFs. This perfect eclipsing along the c-axis (3.27 Å) is crucial as it facilitates effective interaction between the 2D sheets via p-p interactions, which is essential for through-plane charge transport. Such stacking is comparable to that observed in other conductive MOFs, like M_3_(HHTT)_2_, suggesting a shared design principle among high-performance 2D cMOFs. Figure 3d,e shows cryogenic high-resolution transmission electron microscopy (cryo-HRTEM) images of Cu_3_(HITT)_2_ and Ni_3_(HITT)_2_, which are clearly hexagonal and have long-range order. The Fast Fourier Transform (FFT), as shown in the inset of Figure 3e, consistent with a-b lattice parameters between PXRD and FFT, identifies the period of the lattice (~2.3 nm). Figure 3g shows the excellent fringe periodicity of 2.3 nm. Figure 3h–l presents a comparative study of the charge-transport properties of two isostructural 2D electrically conductive metal–organic frameworks (2D-e-MOFs), namely Ni_3_(HITAT)_2_ and Ni_3_(HITBim)_2_ [52]. It provides a compelling visual summary, demonstrating that small changes in molecular design can significantly impact the electrical properties of MOFs, both conceptually and practically. The electronic band structures of Ni_3_(HITAT)_2_ and Ni_3_(HITBim)_2_ are computed and shown in Figure 3h,i. Ni_3_(HITAT)_2_ exhibits broader band dispersion, especially along the in-plane paths (K-G-M), indicating strong electronic coupling and charge-carrier delocalization within the layers. In comparison, Ni_3_(HITBim)_2_ shows a narrower in-plane band gap, with flatter bands near the Fermi level. This indicates that charge mobility inside the planes is likely limited due to the nitrogen-rich HATBim ligand’s more electron-deficient and localized nature. The band gap of Ni_3_(HITBim)_2_ is 0.40 eV, notably larger than 0.21 eV for Ni_3_(HITAT)_2_, aligning with the differing transport behaviors observed. Figure 3j,k shows the diffuse reflectance spectra and corresponding Tauc plots of Ni_3_(HITAT)_2_ and Ni_3_(HITBim)_2_. The Tauc plots show optical band gaps of 0.68 eV for Ni_3_(HITAT)_2_ and 0.85 eV for Ni_3_(HITBim)_2_, confirming the wider electronic band gap in the latter. This further confirms that the electron-deficient HATBim ligand has a minor contribution to the conduction pathway, limiting intraplane transport. Figure 3l shows the room temperature current density versus electric field curves for both MOFs with a two-point probe configuration. The conductivity of Ni_3_(HITAT)_2_ is 44 mScm^−1^, which is an order of magnitude greater than that 0.5 mScm^−1^ for Ni_3_(HITBim)_2_. This nearly two orders-of-magnitude difference clearly illustrates the influence of ligand electronic structure on charge mobility. Therefore, these results highlight the importance of ligand design and electronic structure engineering in fine-tuning the charge transport properties of 2D-e-MOFs. Fascinatingly, the significant difference in conductivity and activation energies between the two MOFs confirms the modulating effect of nitrogen incorporation and the type of p-conjugation pattern on charge delocalization. Ni_3_(HITBim)_2_ specifically shows a greater surface area and porosity, but its electronic transport is significantly impaired because of limited band dispersion and weak orbital overlap.

Therefore, it is concluded that the evolution of MOFs from traditional mono-metallic frameworks to more complex multicluster and multiligand systems highlights the increasing need for functional specificity and design flexibility in gas sensing. Incorporating conductive 2D frameworks, hierarchical porosity, and defect engineering has improved charge mobility and adsorption kinetics, while also enabling precise analyte recognition across various environmental conditions. Although modularity and reticular chemistry provide accurate control over pore size and topology, the main challenge remains in transforming these features into scalable, durable sensors. A solid understanding of structure–property relationships, as shown by the NU-1000 and NU-110x series, is crucial for customizing MOFs to meet specific application needs in sensor devices.

## 3. MOFs Strategies for Gas Sensing

While Section 2 covers the fundamental aspects of Metal–organic frameworks (MOFs) structure and properties, this section emphasizes advanced design strategies to improve gas-sensing performance, including the incorporation of functional groups, hybridization, and morphology control.

### 3.1. H_2_S Sensing

MOFs have emerged as transformative materials in the development of gas sensors, particularly for detecting toxic and environmentally hazardous gases, such as hydrogen sulfide (H_2_S). This section compares various MOFs-based sensing platforms, highlighting structural and material differences, as well as hybrid sensing approaches that enhance H_2_S detection performance. Morphology, metal content, heterojunction design, and synthesis methods also play a role in optimizing sensors for low-power, real-time environmental monitoring.

A highly sensitive, low-temperature H_2_S gas sensor based on a ZnO-CuO composite was synthesized using a low-temperature bimetallic MOF, Zn/Cu-BTC [53]. It was produced through solvothermal synthesis followed by calcination of calcium, resulting in an octahedral ZnO/CuO structure with a well-dispersed p-n junction between p-type CuO and n-type ZnO. By optimizing the Cu:Zn ratio to 1:0.33, the sensor achieved excellent performance, with a sensitivity of 393.35 to 10 ppm H_2_S at a low operating temperature of 40 °C. The device showed a low detection limit of 300 ppb, a rapid response time, and high selectivity against common interfering gases. It also exhibited high repeatability over six cycles, with a performance decline of less than 5% over a 42-day period. Additionally, Joule heating enabled the sensor to recover its sensing ability through electrical pulse desorption, thereby preventing the formation of CuS. The sensor’s ability to detect environmentally relevant gases, such as H_2_S, underscores the significance of MOF-derived composites in developing efficient, low-power, and low-temperature gas sensors suitable for environmental monitoring of hazardous gases. On the other hand, a metal–organic framework (MOF)-based sensor, featuring a CuO/ZnO hollow nanocage, exhibits high sensitivity and rapid response in detecting hydrogen sulfide (H_2_S) [54]. The CuO/ZnO sensor (M3) achieved a sensitivity of 3423 to 10 ppm H_2_S at just 115 °C, with a response time of only 10 s. This enhanced performance is attributed to the hollow nanocage structure, increased oxygen vacancies, and p-n heterojunctions formed between CuO and ZnO. These structural and electronic features promote efficient electron transfer and gas diffusion, thereby improving selectivity, stability, sensitivity, and the detection limit to 100 ppb. Compared to existing materials, the CuO/ZnO sensor performs exceptionally well under mild conditions, marking a breakthrough in metal oxide semiconductor-based gas sensing. This research offers a promising route for developing effective sensors to ensure industrial safety and monitor environmental hazards. Moreover, a new H_2_S gas sensor utilizing cataluminescence (CTL) has been developed, employing MOFs as sensing materials [55]. The study assesses four MOFs, such as MIL-100(Fe), MIL-101(Cr), Zn_3_(BTC)_2_, and ZIF-8. These MOFs are known for their high sensitivity, stability, and rapid response and recovery times, with Zn_3_(BTC)_2_.12H_2_O and ZIF-8 demonstrating particularly outstanding performance. Notably, ZIF-8 detects H_2_S at 3.0 ppm and remains structurally stable after CTL testing. The findings underscore the importance of metal ions in the CTL process, showing that zinc-based MOFs have inherent advantages for H_2_S sensing. Furthermore, a high-performance H_2_S gas sensor was developed using a bamboo-like CuO/In_2_O_3_ heterostructure based on MOFs [56]. Using Cu^2+^ with CPP-3(In) (In-MOFs) as a precursor, the composite achieved an optimized CuO content of 3.5 wt% and formed effective p-n heterojunctions. The sensor demonstrated excellent sensitivity to H_2_S with a response of 8.5 times that of pristine In_2_O_3_ at 5 ppm concentration and a detection limit of 200 ppb at 70 °C, which is due to increased surface area, p-n junction formation, a high concentration of chemisorbed oxygen, and improved chemical reactivity between CuO and H_2_S. Interestingly, a highly sensitive H_2_S gas sensor has been developed using γ-Fe_2_O_3_/rGO composites based on MIL-88 metal–organic frameworks, which functions effectively at room temperature [57]. The addition of rGO enhanced electron transmission and dispersion of γ-Fe_2_O_3_ octahedrons, resulting in an exceptional response (R_air_/R_gas_ = 520.73 at 97 ppm H_2_S) and high selectivity against interfering gases, such as NH_3_, NO, and SO_2_. This enhanced performance stems from a surface-controlled sensing mechanism leveraging conductivity and abundant active sites in rGO. The study demonstrates that the metal oxides and 2D materials derived from MOFs can advance low-temperature gas sensor technology. Moreover, a significant advancement in the sensing of H_2_S gas has been realized using a CuO-decorated ZnO sensor, which was prepared using the metal–organic framework ZIF-8 [58]. Its most important innovation is that it achieves an ultra-high response of 941 to 10 ppm H_2_S at a relatively low working temperature (175 °C), which is 80 times greater than that of pristine ZnO. This enhancement is a synergistic combination of a p-n heterojunction between n-type ZnO and p-type CuO, more oxygen vacancies, and stronger chemisorption. The sensor exhibits enhanced sensitivity, selectivity, and efficiency compared to existing H_2_S sensors. Using MOF-derived ZnO as a high-surface-area scaffold and sensitizing it with CuO provides an economical and practical approach for H_2_S monitoring. Interestingly, creating a room-temperature gas sensor demonstrates that a bimetallic metal-MOFs [MOF-919(FeCu)] provides the highest efficiency in detecting H_2_S [59]. It is innovative because it features a two-metal system with Fe and Cu within the same MOF structure, where this dual functionality boosts both electrochemical and adsorption capabilities. MOF-919 achieved the highest response (931.6% at 10 ppm H_2_S), the lowest detection limit (0.31 ppm) for coal samples compared to monometallic MOFs, MIL-100(Fe), and HKUST-1(Cu), and showed high selectivity in a mixed-gas environment. DFT calculations supported these findings by indicating that H_2_S adsorption energies at Fe and Cu sites were stronger. MOF-919 also demonstrated excellent repeatability and stability, even in humid conditions. This combined theoretical and experimental work establishes MOF-919 as a key material for low-power, room-temperature H_2_S sensing and points toward promising strategies for designing future sensors with dual-metal active sites. Conversely, a new method improves gas detection using metal oxides from MOFs, notably CuO, NiO, and ZnO, which are effective for sensing H_2_S, CO, and H_2_ gases, respectively [60]. The innovative aspect lies in synthesizing MOFs intentionally and calcining them at optimal temperatures (400–600 °C) to produce porous, nanostructured metal oxides with high surface area and specific morphology. This thermochemical process preserved the porosity of the original MOF and significantly improved electrical conductivity and gas responsiveness. Notably, all sensors demonstrated high sensitivity, stability, and selectivity at elevated temperatures: CuO for H_2_S, NiO for CO, and ZnO for H_2_. Moreover, a new, highly effective H_2_S gas sensor has been developed using a flexible mixed-matrix membrane. This membrane integrates MOF-5 microparticles within a chitosan/glycerol (CS/IL) polymer matrix [61]. The sensor performs exceptionally well at room temperature, with a low detection limit of 1 ppm, which is crucial for practical applications. It features a quick reaction time (<8 s), rapid recovery (<30 s), and maintains 97% of its response over 21 days, demonstrating long-term stability. The key innovation lies in the synergistic combination of MOF-5′s high porosity and selectivity with the proton-conductive CS/IL matrix, enhancing gas adsorption and proton transport. Additionally, the sensor operates at an ultra-low bias voltage (120 mV) and consumes minimal power. Furthermore, a significant improvement in gas sensing has been achieved with the development of a ratiometric luminescent probe for detecting hydrogen sulfide (H_2_S), utilizing a functionalized MOFs [62]. The innovation lies in the postsynthetically modified UiO-66-(COOH)_2_, which incorporates Eu^3+^ and Cu^2+^ ions, enabling the selection of dual luminescent emissions, a sharp Eu^3+^ emission, and ligand-centered (LC) emission. When treated with H_2_S, the high affinity of sulfide ions to Cu^2+^ ions overturns the Cu^2+^ induced quenching of Eu^3+^ in Eu^3+^ solution, thereby producing a much intense signal. This bimolecular emitting structure enables self-calibrated ratiometric sensing, which is less prone to environmental interference, a typical problem of single-emission probes. Furthermore, the sensor exhibits high selectivity and a rapid response time of 30 s, performing well under physiological conditions. The decomposition of the logic gate behavior offers a conceptual advantage, indicating that the MOF-based platform is a promising and flexible instrument for real-time, selective H_2_S detection. Interestingly, a significant breakthrough in gas sensing has been achieved with the development of a new platform that detects hydrogen sulfide (H_2_S) using a flexible MOF-based mixed-matrix membrane (MMM) [63]. The key innovation involves incorporating highly stable Al-MIL-53-NO_2_ nanoparticles into a poly(vinylidene fluoride) (PVDF) matrix, up to 70 wt%. Unlike traditional powder-form MOF sensors, this approach enhances flexibility, mechanical strength, and processability, overcoming issues of fragility and low dispersibility. The resulting MMM exhibits excellent H_2_S detection capabilities, including fluorescence, high selectivity against interferents, and a minimum detectable limit of 92.31 nM, which is three orders of magnitude lower than previous powder MOF sensors. This development offers a scalable, sensitive sensing platform, opening new pathways for MOF-polymer hybrids in chemical sensing and environmental monitoring.

**Figure 4:**
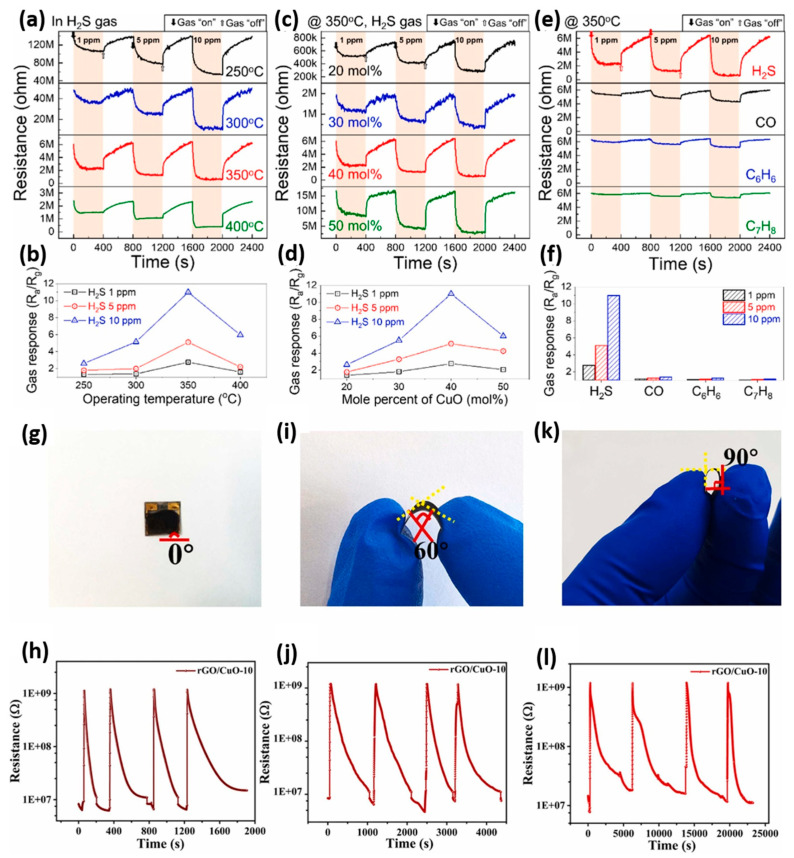
(**a**) Transient resistance measurements of the gas sensor ZnO/CuO (40 mol%) compared to the concentration of 1, 5 and 10 ppm of H_2_S in different operating temperatures (250–400 °C). (**b**) Equivalent gas response–temperature curves at concentrations of 1, 5, and 10 ppm of H_2_S, respectively, showing 350 °C as the ideal operating temperature. (**c**) Transient resistance of ZnO/CuO sensors with different CuO molar ratios (20–50 mol%) on exposure to H_2_S (1–10 ppm) at 350 °C. (**d**) Gas response based on CuO mol% shows that the gas response is better at a concentration of 40 mol% CuO, (**e**) transient resistance measurements of the optimized 40 mol% CuO sensor for various interfering gases, and (**f**) selectivity profile of the sensor showing significantly higher response to H_2_S compared to other gases. Reproduced with permission from Ref. [64]. Copyright (2021) Elsevier. (**g**–**l**) shows the rGO/CuO-10 flexible sensor response–recovery curves (bent or flat) to 10 ppm H_2_S gas at various bending angles (**g**,**h**) flat (0°), (**i**,**j**) bent at 60°, and (**k**,**l**) bent at 90°. The stable reaction across all bending angles also yields a sensor with magnificent mechanical flexibility and stability, making it suitable for real-life applications. Reproduced with permission from Ref. [65]. Copyright (2025) Elsevier.

Figure 4a–f presents a detailed evaluation of the gas sensing performance of ZnO/CuO nanocomposites derived from MOFs, with varying ratios of CuO to ZnO [64]. Figure 4a displays the transient resistance behavior of the ZnO/CuO (40 mol%) gas sensor across different H_2_S concentrations (1, 5, and 10 ppm) and operating temperatures (250–400 °C). The resistance sharply decreases with increasing temperature, typical of an n-type semiconductor, indicating that ZnO primarily influences the sensing response. Figure 4b shows the maximum response at 350 °C, where the balance between H_2_S adsorption and desorption rates is optimal. Figure 4c shows the resistance variation in sensors with different amounts of CuO (20–50 mol%) in response to H_2_S at 350 °C. A quantitative comparison of the response of gas in various compositions is shown in Figure 4d. Its response is highest in the 40 mol% CuO sensor due to the optimal formation of p-n heterojunctions between ZnO and CuO. Both underloading and overloading of CuO disrupt this balance, reducing the number of heterojunctions and consequently lowering the sensing performance. The essential sensor property, selectivity, is illustrated in Figure 4e,f. The selectivity of the ZnO/CuO (40 mol%) sensor is high, as it produces a stronger signal for H_2_S compared to CO, benzene (C_6_H_6_), and toluene (C_7_H_8_). The strong response to H_2_S is further enhanced by the conversion of CuO into conductive CuS when exposed to H_2_S, which significantly changes the resistance. Therefore, it is concluded that Figure 4a–f confirm that the sensing of H_2_S gas by nanocomposites of ZnO/CuO with 40 mol% CuO and an operating temperature of 350 °C are most effective, as the sensitivity, selectivity, and optimum operating conditions are achieved. MOF-based and MOF-derived materials show significant performance improvements worth exploring, but several challenges still need to be addressed. These include ensuring long-term stability in various environmental conditions, achieving reproducibility across sensors, and developing scalable fabrication methods. Despite the promising performance benefits of MOF-based materials, issues such as environmental stability, reproducibility, and scalable manufacturing persist. On the other hand, Figure 4g–l illustrates the response-recovery behavior of the rGO/CuO-10 sensor under mechanical strain at bending angles of 0°, 60°, and 90° [65]. These graphs are crucial for demonstrating the sensor’s mechanical durability and flexibility, which are the key features for wearable and portable sensing devices. Notably, the response patterns remain highly consistent across all bending states, indicating that the sensor’s performance is largely unaffected by physical deformation. Figure 4g,h shows that the sensor yields stable, reproducible results up to 10 ppm of H_2_S at 0°, making it the baseline against which comparative analysis can be conducted. Furthermore, with subsequent bends of 60°, as shown in Figure 4i,j and 90°, as illustrated in Figure 4k,l, the sensor exhibits comparable response and recovery times and intensity. The combined effect of integrating rGO enhances sensing stability under deformation, while also boosting electrical conductivity, surface area, and mechanical strength. Unlike metal oxide-based sensors, especially MOF-derived types, increasing flexibility is challenging, making rGO/CuO-10 mixtures promising for flexible electronics and environmental monitoring devices.

### 3.2. Humidity Sensing

Recent progress in humidity sensing technology has centered on MOFs due to their customizable porosity, large surface area, and versatile functions, resulting in the creation of highly sensitive and durable humidity sensors. Researchers have discovered new benefits for using mesoporous nanostructures as humidity sensors. For instance, a team led by Zhang developed a 3D mesoporous Co_3_O_4_ hollow polyhedron that demonstrated high sensitivity and stability when exposed to changing humidity levels [66]. Metal–organic framework-based nanostructures show great promise for use in wearable and environmental sensing applications. In addition, the humidity-sensing capabilities of MOF-derived polyelectrolyte films, especially those based on UIO-66, exhibit remarkable potential [67]. Among the tested sensors [P0 (0% 2-SO_3_Na-BDC), P1 (10% 2-SO_3_Na-BDC), P2 (20% 2-SO_3_Na-BDC), P3 (0% 30-SO_3_Na-BDC), and P4 (40% 2-SO_3_Na-BDC)], the P3 sensor with sulfonated UIO-66 derivatives stood out for its high humidity sensitivity and quick response. This is likely due to sulfonate groups and structural defects that enhance water molecule adsorption and desorption. The sensor responded rapidly, with times of 3.1 s for response and 1.5 s for recovery, surpassing many previous MOF-based sensors for humidity detection. Additionally, the impedance response of the P3 sensor exhibited very low humidity hysteresis (~1.2% RH), indicating excellent reversibility and consistent sensing performance. The porous structure and hydrophilic properties of the sensing material enable the formation of conductive pathways via adsorbed water layers at high humidity levels, resulting in significant impedance changes. Notably, the P3 sensor demonstrated consistent performance over repeated cycles and extended use (30 days), highlighting its stability and suitability for practical applications. Supportive evidence from complex impedance spectroscopy and QCM studies confirmed the sensing mechanism, showing that both framework defects and synergistic sulfonate-channel interactions promote rapid ion transport and water exchange. This affirms a rational design approach for creating fast, reliable, and thermally stable humidity sensors through MOF-derived polyelectrolyte films. Additionally, employing IL-modified MOFs, especially UIO-66, is an effective method for sensing low humidity [68]. Adding hydrophilic IL ligands into the MOF structure greatly boosts water adsorption by creating defect sites and polar groups that promote hydrogen bonding with water molecules. Among the tested sensors, P3 (30% IL content) exhibited the best overall performance, characterized by quick response and recovery times (0.6 s/1.7 s), low humidity hysteresis (~0.2% RH), and good linearity over the 5–30% RH range. Compared to earlier materials, IL/MOF sensors offer better sensitivity and stability at ultra-low humidity, which is vital for applications in microelectronics and environmental monitoring. The evenly dispersed IL prevented aggregation, ensuring consistent film quality and reliable transmission of the humidity signal. The sensors also maintained stable impedance over multiple cycles and showed little performance loss after long exposure to high humidity, demonstrating their durability. This research demonstrates that combining chemical coordination with photo-induced polymerization can enhance the hydrophilicity and structure of MOF films, offering a scalable approach for high-performance gas sensors that can detect trace moisture levels. Additionally, a novel MIL-101(Cr)-based humidity sensor shows outstanding performance, making it a promising material for sensing applications [69]. The porous crystalline structure of MIL-101(Cr) provides a high specific surface area (~2292 m^2^/g), enabling effective adsorption and diffusion of water molecules. The MIL-101(Cr)-based humidity sensor exhibits a significant impedance change of over three orders of magnitude across a wide relative humidity (RH) range (33–95%) at 100 Hz, indicating high sensitivity. It also responds quickly, with a response time of 17 s and a recovery time of 90 s, due to the efficient adsorption–desorption dynamics facilitated by the MOF’s high porosity. On the other hand, MOFs are highly versatile materials used in gas sensing, including humidity detection, due to their high porosity, large surface area, and adjustable chemistry [70]. The effectiveness of MOF-based sensors depends mainly on analyte molecules, which interact with the MOF, affecting the sensor’s electrical or optical signals. For humidity sensing, MOFs offer remarkable sensitivity and quick response and recovery times, because of their hydrophilic groups and customized pore structures. MOF-based sensors of impedance, capacitance, and resistance demonstrate varied responses at different humidity levels, with some achieving response times as fast as 0.4 s and detection limits below 1% RH. Nonetheless, challenges, such as film uniformity, conductivity, and durability in environmental conditions, still exist. Overall, the gas sensing abilities of MOF-based humidity sensors hold great promise for industrial, environmental, and wearable uses, highlighting the need for further research into structure–function relationships and material engineering. Interestingly, MOF-coated textile sensors, originally designed for humidity detection, also show promising capabilities in gas sensing [71]. Using the Langmuir–Blodgett (LB) technique, MIL-96(Al) nanoparticles were uniformly deposited onto interdigitated electrodes embroidered into cotton and linen fabrics. The linen-based TEX sensor exhibited high sensitivity and selectivity to humidity, with improved MOF film coverage resulting in a low detection limit of 0.71% RH and a linear response across a wide humidity range. These sensors maintained stability and repeatability, with only about 16% signal loss after three weeks in ambient conditions. Overall, these findings highlight the potential of textile-based MOF humidity sensors combined with advanced gas sensors as reliable, reusable, and selective options for environmental monitoring and wearable applications. These advancements collectively highlight the transformative potential of MOF-based humidity sensors. They include polyelectrolyte films, IL-integrated frameworks, and textile-integrated devices, all paving the way for next-generation sensors that are highly sensitive, durable, and multifunctional. These sensors are designed for a variety of real-world applications, including environmental monitoring, healthcare, and smart wearables.

**Figure 5:**
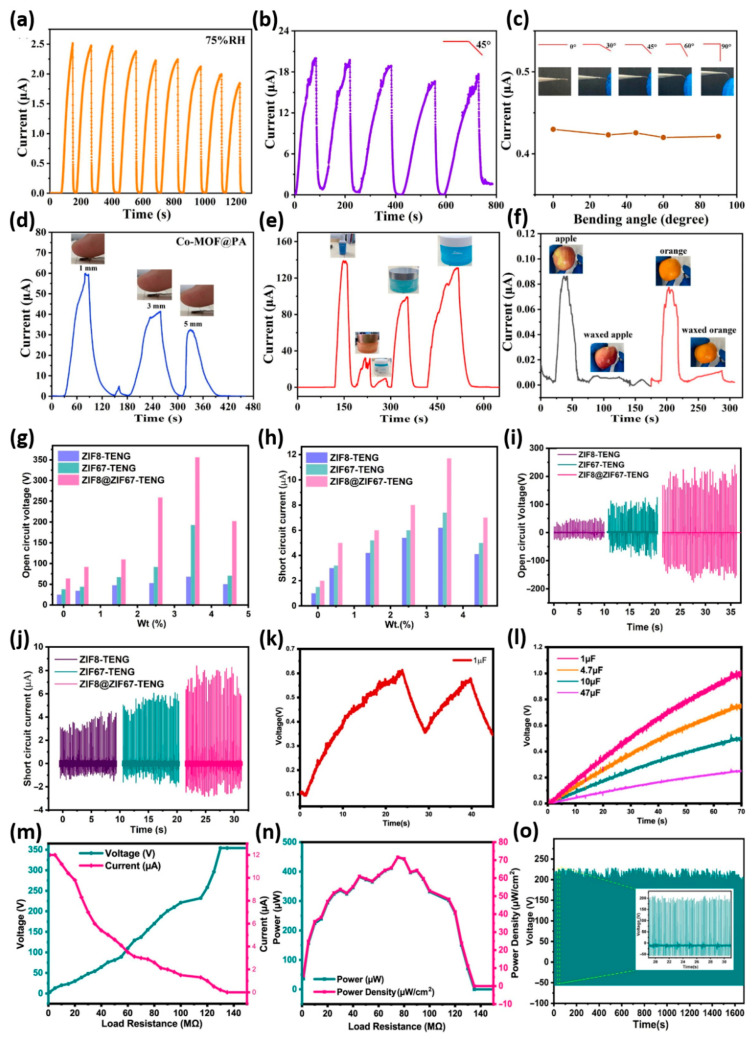
Multifunctional performance and mechanical robustness of the flexible Co-MOF@PA-based humidity sensor. (**a**) Repeatability of the sensor across nine consecutive response-recovery cycles at 75% RH, demonstrating excellent stability, (**b**) response and recovery behavior of the flexible sensor under repeated bending at a 45° angle, (**c**) initial current values of the sensor under various bending angles, indicating mechanical durability and electrical stability, (**d**) non-contact sensing performance as a moist fingertip approaches the sensor at varying distances (1–5 mm), highlighting sensitivity to ambient humidity gradients, (**e**) evaluation of the moisturizing effects of five commercial cosmetic products applied to human skin, showing differential responses based on hydration levels, and (**f**) humidity response comparison between waxed and unwaxed apples and oranges, demonstrating the sensor’s capability for freshness monitoring through moisture emission detection. Reproduced with permission from Ref. [72]. Copyright (2022) Elsevier. (**g**–**o**) plays a pivotal role in establishing the superior triboelectric performance of the ZIF8@ZIF67-based triboelectric nanogenerator (TENG) relative to its parent counterparts, ZIF-8 and ZIF-67. (**g**,**h**) Comparison of open-circuit voltage (V_oc_) and short-circuit current (I_sc_) output at varying weight percentages (0–4.5 wt%) for ZIF8-TENG, ZIF67-TENG, and ZIF8@ZIF67-TENG, showing optimum performance at 3.5 wt%, (**i**,**j**) time-resolved V_oc_ and I_sc_ outputs over a 10 s interval, demonstrating consistent and superior electrical performance of ZIF8@ZIF67-TENG compared to other, (**k**) charge–discharge cycles of a 1 µF commercial capacitor powered by ZIF8@ZIF67-TENG, highlighting repeatable charging ability, (**l**) charging profiles of different capacitors (1, 4.7, 10, and 47 µF), indicating the scalability and efficiency, (**m**) output voltage and current versus varying external load resistance, demonstrating increasing voltage and decreasing current trend, (**n**) calculated power density and instantaneous power across different load resistances, with a maximum of 460 μW (736 mW/m^2^) at 75 MΩ load, and (**o**) long-term operational stability of ZIF8@ZIF67-TENG sensor under 10,080 mechanical cycles, confirming its high durability and robustness. Reproduced with permission from Ref. [73]. Copyright (2025) Elsevier.

Figure 5a–f illustrates the multifunctional performance and mechanical stability of the flexible Co-MOF@PA-based humidity sensor (phytic acid (PA) modified on the surface of Co-MOF nanosheets), highlighting its utility in real-world, wearable, and non-contact sensing applications [72]. Figure 5a,b demonstrates the repeatability of the sensor across nine consecutive humidity sensing cycles at 75% relative humidity (RH), showcasing its reproducible response with negligible drift or baseline instability. This characteristic is crucial for consistent performance in wearable or environmental monitoring applications, where long-term reliability is essential. Figure 5c demonstrates the sensor’s mechanical flexibility and ability to tolerate deformation. Even with repeated bending at 45°, or when tested at various angles, the current stays stable, showing strong mechanical and electrical stability. This durability under strain is essential for embedding the sensor into flexible or conformal substrates, such as skin patches or smart textiles. Figure 5d,e further highlights the sensor’s ability to detect humidity without contact. As a moist fingertip gets closer to the sensor (1–5 mm), a clear gradient in the output signal appears, proving the sensor can sense humidity changes without physical touch. This is especially useful in hygienic settings or human–machine interface systems. Additionally, in Figure 5e, the sensor effectively distinguishes the moisturizing effects of different commercial cosmetic products on human skin. This suggests the device could be a valuable tool in dermatology or cosmetic product testing. Finally, Figure 5f shows that the sensor can evaluate fruit freshness by differentiating between waxed and unwaxed apple and orange surfaces. The sensor detects a higher humidity signal for unwaxed fruits, reflecting increased moisture emission. This highlights the Co-MOF@PA-based humidity sensor’s potential for non-invasive freshness monitoring in food storage and retail environments. Overall, Figure 5a–f demonstrates the Co-MOF@PA-based sensor’s versatility, durability, and high sensitivity, confirming its usefulness for wearable electronics, contactless interfaces, and diagnostic applications in environmental and biomedical fields, due to hydrophilicity in humidity detection. On the other hand, Figure 5g–o plays a pivotal role in establishing the superior triboelectric performance of the ZIF8@ZIF67-based triboelectric nanogenerator (TENG) relative to its parent counterparts, ZIF-8 and ZIF-67 [73]. The open-circuit voltage (V_oc_) and short-circuit current (Isc) plots Figure 5g,h as functions of different weight percentages (wt%) clearly show optimal performance at 3.5 wt%. ZIF8@ZIF67-TENG reaches 359 V V_oc_ and 11.7 µA I_sc_. This exceeds the output of ZIF-8 and ZIF-67 alone, demonstrating the effectiveness of the core–shell architecture in boosting charge generation. The time-resolved measurements Figure 5i,j further support this finding by showing consistent and reproducible voltage and current profiles over a 10 s interval. Figure 5k confirms the practical utility of the TENG by demonstrating the effective charging and discharging of a 1 µF capacitor, while Figure 5l highlights the sensor’s scalability through charging behaviors of capacitors of varying capacitances up to 47 µF. Crucially, Figure 5m,n maps the output current and voltage across a wide range of external load resistances, pinpointing a peak instantaneous power output of 460 µW and power density of 736 mW/m^2^ at 75 MΩ, surpassing many previously reported MOF-based polymer-composite TENGs. This exceptional performance is attributed to enhanced surface roughness, higher dielectric constant, and improved charge trapping facilitated by the ZIF8@ZIF67 core–shell design. Lastly, Figure 5o validates the sensor’s operational durability through a 10,080-cycle stability test, demonstrating its robustness for long-term use.

### 3.3. NO_2_ Sensing

Recent progress in MOF-derived and MOF-functionalized materials has revealed significant potential for NO_2_ gas detection across various conditions. Unlike traditional solid-state oxide sensors, these materials offer high surface areas, adjustable porosity, and customizable active sites, allowing precise regulation of adsorption–desorption and charge transfer. Their diverse structures, from nanocrystals within preserved MOFs to heterojunction composites and noble-metal enhanced conductive MOFs, have enabled solutions for specific challenges like low-temperature sensing, humidity resistance, and multi-gas selectivity. Furthermore, we compare recent sensor designs, focusing on their structure, manufacturing methods, and sensing mechanisms, and their impact on key performance metrics such as sensitivity, selectivity, detection limits, response and recovery times, and stability.

The Co_3_V_2_O_8_/La_10_Si5.5Al_0.5_O_26.75_ NO_2_ sensor exhibits excellent gas detection capabilities, due to its MOF-derived microstructure and optimized electrode–electrolyte interface [74]. Operating at 575 °C, it provides high sensitivity (78.2 mV/decade for 50–500 ppm NO_2_), rapid response (47 s at 500 ppm), and consistent linearity across a broad concentration range. The interconnected-rod structure of Co_3_V_2_O_8_, combined with the porous electrolyte layer, increases the three-phase boundary, promoting NO_2_ diffusion and boosting catalytic activity through highly dispersed Co/V sites. Compared to similar sensors made via solid-state methods, the MOF-derived sensor nearly doubles sensitivity and achieves shorter response times, especially at higher NO_2_ levels. Its sensing mechanism is based on the mixed-potential model, involving cathodic reduction of NO_2_ and anodic oxidation of O^2−^ at the triple-phase boundary. These combined structural and electrochemical features make MOF-derived Co_3_V_2_O_8_ a promising electrode for high-temperature NO_2_ detection in challenging environments. On the other hand, the CuO_x_ octahedron-based NO_2_ sensor, formed from Cu-based MOFs, shows outstanding gas sensing features characterized by a temperature-dependent p-n transition [75]. From 25 to 180 °C, it behaves as a p-type sensor because NO_2_ adsorption captures electrons from acceptor levels and reacts with surface oxygen, increasing hole concentration and lowering resistance. Above 200 °C, the behavior switches to n-type, as a greater coverage of O^−^ species reacts with NO_2_, releasing electrons, reducing hole concentration, and increasing resistance. This reversible switching allows qualitative NO_2_ detection even in mixed gases, without relying on response magnitude comparisons. The sensor exhibits a high response of 76.7% at 25 °C for 500 ppb NO_2_, with fast adsorption kinetics and excellent repeatability across cycles. It maintains performance across different humidity levels, with minimal interference, and shows linear or saturated responses depending on concentration. The porous, high-surface-area structure enhances gas diffusion and adsorption, and the temperature-tunable selectivity offers a promising strategy for environmental monitoring applications. The SnO_2_-M-OV-300 sensor, prepared from Sn-MOFs via thermal treatment, shows outstanding NO_2_ sensing capabilities due to its unique structural and electronic features [76]. By preserving the MOFs while embedding SnO_2_ nanocrystals, it offers a high surface area, numerous porous channels, exposed Sn active sites, and a high density of oxygen vacancies. These characteristics enhance NO_2_ adsorption, facilitate quick diffusion, and improve charge transfer. The sensor delivers an ultrahigh response of 11,677 to 1 ppm NO_2_ at 120 °C, roughly 100 times greater than pure SnO_2_ and still shows a significant response (170) at 50 °C, with a detection limit as low as 1 ppb. It features rapid response and recovery times (33 s/15 s), excellent selectivity against typical VOCs, and maintains stability and reproducibility over cyclic testing. DFT studies indicate that oxygen vacancies and Sn atoms serve as active adsorption sites, with vacancies showing stronger NO_2_ affinity and more charge transfer capacity. Combined experimental and theoretical insights demonstrate that defect engineering and MOF-based nanostructuring are effective approaches for creating high-performance, low-temperature gas sensors suitable for real-time environmental monitoring. Moreover, pristine SnO_2_ nanowires (NWs) and MOF-901-coated SnO_2_ NWs show different gas sensing behaviors under various humidity levels [77]. Both sensors detect NO_2_ best at 200 °C; pristine SnO_2_ NWs respond more strongly in dry air (9.7 response to 10 ppm NO_2_) than MOF-901-coated NWs (8.2 response), because of its larger active surface. Humidity affects both sensors: at 80% RH, pristine NW’s response drops to 4.0, while MOF-901-coated NWs still respond at 6.6, retaining about 80% of dry-air sensitivity. This improvement is due to MOF-901’s hydrophobicity (WCA = 123°) and its function as a molecular sieve, which reduces water adsorption and enhances NO_2_ interaction. Testing shows both sensors prefer NO_2_ over other gases, but MOF-901-coated NWs perform better in humid conditions. The sensing mechanism relies on NO_2_’s electron withdrawal, which increases resistance by narrowing conduction pathways and modulating Schottky barriers. Overall, coating with MOF-901 provides more stable, selective NO_2_ detection in high humidity, overcoming a key limitation of uncoated metal oxide sensors. On the other hand, the gas sensing capabilities of the Au_3_@WS_2–_2/Ni_3_(HITP)_2_ flexible chemiresistive sensor show notable improvements through dual functionalization [78]. Ni_3_(HITP)_2_, an n-type conductive MOF, naturally detects NO_2_ at room temperature but has limited sensitivity. Adding 2 mol% WS_2_ quantum dots (QDs) forms p-n heterojunctions, increasing active sites and aiding charge separation. Meanwhile, 3 mol% Au nanoparticles (NPs) create Schottky junctions, enhancing NO_2_ adsorption and desorption. This combined effect leads to high response (14.73% to 5 ppm NO_2_), quick response/recovery times (97 s/110 s), and detection limits below 0.5 ppm at approximately 25 °C. The sensor shows strong selectivity for NO_2_ over gases, such as H_2_, H_2_S, CO, and CH_4_, maintains performance across varying humidity, and demonstrates excellent repeatability over multiple cycles. Mechanical tests reveal minimal degradation (<9.3%) after 500 bending cycles, indicating suitability for wearable use. These results highlight that strategic noble metal/TMD co-functionalization of conductive MOFs is an effective approach for high-performance, flexible NO_2_ sensing at room temperature. Fascinatingly, the V-MOF_120_(PTA)-based NO_2_ gas sensor exhibits outstanding sensing capabilities, making it highly suitable for advanced applications [79]. Created through a one-step hydrothermal process and combined with laser-induced graphene electrodes, it achieved an impressive average response of approximately 800.8% at 100 ppm NO_2_, with a detection limit as low as 1 ppm. Its quick response and recovery times (230 s/39.8 s) indicate efficient adsorption–desorption dynamics, due to its high specific surface area (28.2 m^2^/g) and numerous active sites. The sensor proved highly repeatable, showing consistent results over multiple cycles, and maintained long-term stability with minimal signal loss over 30 days. Selectivity tests showed little interference from ethanol, methanol, acetone, and ammonia, due to NO_2_’s higher electron affinity, which leads to significant charge transfer and resistance changes. However, performance drops sharply at relative humidity levels above 70%, as water molecules compete for active sites, decreasing sensitivity. Mechanistically, NO_2_ adsorption on the V_2_O_5_-rich surface increases the electron depletion layer thickness, raising resistance, while desorption returns it to baseline. Compared to other vanadium-based sensors, V-MOF_120_(PTA) offers superior sensitivity at room temperature without external heating, highlighting its promise for low-power, portable environmental monitoring. Interestingly, the ZnO/ZnWO_4_ nanoparticle-based gas sensor showed excellent NO_2_ detection capabilities, due to the synergistic effects of p-n heterojunction formation and optimized composition [80]. Incorporating ZnWO_4_ into ZnO, derived from polyoxometalate/metal–organic framework (POM/MOF) precursors, greatly improved electron–hole separation, reduced recombination, and increased charge carrier mobility. This resulted in a maximum response of 26.39 at 1 ppm NO_2_ and 140 °C, which is 7.26 times higher than pure ZnO. The heterojunction enabled efficient electron transfer from ZnO to ZnWO_4_, creating a thicker electron depletion layer upon NO_2_ adsorption and enhancing resistance changes. It achieved a detection limit of 4.32 ppb and showed excellent repeatability, stability over 30 days, and a linear response to concentration changes. However, high humidity significantly reduced the response because of water adsorption at active sites. These findings suggest that POM/MOF-derived ZnO/ZnWO_4_ composites are promising for low-temperature, high-sensitivity NO_2_ detection, though future work should address humidity interference for practical use.

**Figure 6:**
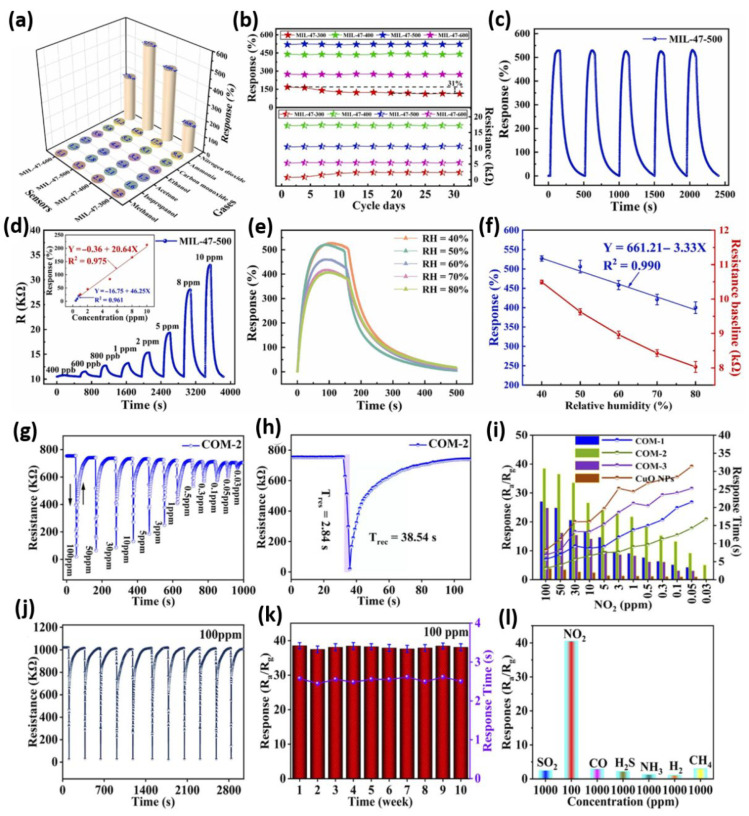
(**a**–**f**) Gas sensing performance of MIL-47-derived porous V_2_O_5_ sensors. (**a**) Selectivity toward various gases (10 ppm) at 150 °C. (**b**) Long-term stability over 30 days toward 5 ppm NO_2_ at 150 °C. (**c**) Repeatability toward 5 ppm NO_2_ at 150 °C. (**d**) Dynamic response-recovery curves and calibration plots for NO_2_ concentrations from 400 ppb to 10 ppm at 150 °C. (**e**) Effect of relative humidity (RH) on NO_2_ response (5 ppm) at 150 °C. (**f**) Influence of RH on baseline resistance. Reproduced with permission from Ref. [81]. Copyright (2023) Elsevier. (**g**–**l**) Gas-sensing performance of metal–organic framework (MOF)-derived CuO NPs/Ti_3_C_2_T_x_ MXene composites towards NO_2_ at room temperature. (**g**) Dynamic response–recovery curves of COM-2 over NO_2_ concentrations ranging from 100 to 0.03 ppm. (**h**) Enlarged response curve for 100 ppm NO_2_, showing rapid response and recovery with high sensitivity. (**i**) Sensitivity comparison of pure CuO NPs and COM-1, COM-2, and COM-3 composites, highlighting COM-2’s superior performance and low detection limit (30 ppb). (**j**) Repeatability test over 10 consecutive cycles for COM-2 at 100 ppm NO_2_. (**k**) Long-term stability over 10 weeks. (**l**) Selectivity of COM-2 towards NO_2_ against common interfering gases at 1000 ppm, illustrating strong NO_2_ preference due to synergistic heterojunction effects, abundant active sites, and enhanced charge transfer. Reproduced with permission from Ref. [82]. Copyright (2024) Elsevier.

Figure 6a–f comprehensively evaluates the MIL-47-derived porous V_2_O_5_ sensors in terms of selectivity, stability, repeatability, detection limit, and humidity tolerance [81]. Figure 6a illustrates that all sensors favor NO_2_ detection at 150 °C, due to the high electron affinity of 2.3 eV of NO_2_, which enhances charge transfer. This is in contrast to gases like NH_3_ (0.16 eV) and VOCs, which need higher activation temperatures. In Figure 6b, the long-term stability results show that MIL-47-500 maintains a consistent baseline and response over 30 days. Meanwhile, MIL-47-300 experiences about a 31% decline and increased resistance, which is caused by residual organics and poor crystallinity. Figure 6c shows excellent repeatability for MIL-47-500, with consistent responses over five NO_2_ exposure cycles. Figure 6d presents dynamic response curves across a concentration range from 400 ppb to 10 ppm at 150 °C. Even at sub-ppm levels, responses are clearly detectable, such as 2.5% at 400 ppb, and the fitting curves reveal distinct linearity in both low-ppb and ppm ranges. The detection of limit (LOD) is calculated at 400 ppb, indicating high sensitivity. Figure 6e,f illustrates the relative humidity (RH) influence on the response of the sensor. As RH increases, the response decreases because water competes for active sites, blocking them. At the same time, baseline resistance drops since water releases electrons through reactions with adsorbed oxygen, raising conductivity. Quantitatively, a 1% change in RH produces a response change comparable to a 0.5 ppm shift in NO_2_, emphasizing the importance of controlling humidity during practical use. On the other hand, Figure 6g–l illustrates the gas-sensing performance of the CuO NPs/Ti_3_C_2_T_x_ MXene composites, especially COM-2, toward NO_2_, demonstrating its effectiveness at room temperature [82]. Figure 6g presents the transient response of COM-2 across a wide NO_2_ concentration range (100–0.03 ppm), showing a sharp resistance decrease when exposed to gas and effective recovery in air, indicating p-type behavior. Figure 6h zooms in on the response at 100 ppm, highlighting an excellent response and recovery times (T_res_ = 2.84 s, T_rec_ = 33.5 s) along with high sensitivity (S = 38.54). Figure 6i compares the sensing performance of CuO NPs, COM-1, and COM-3, clearly showing that COM-2 has the highest sensitivity and a detection limit as low as 30 ppb. Repeatability and stability are demonstrated in Figure 6j,k, where COM-2 maintains a consistent response over 10 consecutive adsorption–desorption cycles and sustains performance for 10 weeks, indicating structural robustness and stable active sites. Figure 6l emphasizes the outstanding selectivity of COM-2, showing a markedly higher response to NO_2_ compared with SO_2_, CO, H_2_S, NH_3_, H_2_, and CH_4_, even when these interfering gases are tested at higher concentrations (1000 ppm). This selectivity is attributed to the synergistic effects of abundant oxygen vacancies, high surface area, MOF, paramagnetic CuO properties, and the electrophilic nature of NO_2_ molecules, which enable preferential adsorption and electron transfer at room temperature. Interestingly, Figure 6g–l confirms that the optimized COM-2 architecture comprising MOF-derived octahedral CuO nanoparticles anchored on conductive MXene nanosheets creates numerous heterojunction interfaces, promotes carrier migration, and offers abundant chemisorption sites. This synergy improves adsorption–desorption dynamics, response strength, and stability, positioning COM-2 as a top candidate for high-performance NO_2_ detection in ambient conditions. The results strongly indicate that designing CuO/MXene heterostructures can surpass the limitations of individual components, facilitating practical, low-power environmental monitoring solutions. Therefore, future research should focus on integrating these advanced sensing architectures into low-power, compact, and potentially wearable devices, as well as developing multi-functional platforms capable of detecting multiple hazardous gases simultaneously in real-world environments. These efforts are crucial to transform laboratory innovations into reliable, commercially viable NO_2_ monitoring solutions.

### 3.4. VOCs Sensing

Acetone is one of the key volatile organic compounds (VOCs) in human breath, serving as a non-invasive indicator for conditions like diabetes, ketosis, and issues with fat metabolism. Detecting acetone accurately is challenging due to several factors, including its low concentration in the body, interference from other VOCs like ethanol and isopropanol, and the impact of humidity on breath analysis. Recent research has focused on developing heterostructures, porous nanomaterials, and MOF-derived composites, which offer high surface areas, adjustable chemistry, and catalytic properties. These strategies aim to provide ultra-sensitive detection, quick response and recovery, and stable performance in humid conditions. However, various MOF-based or MOF-enhanced sensing platforms improve acetone detection through different material design approaches. The MOF-derived SnO_2_-ZnO heterostructure shows improved chemiresistive acetone detection compared to pure ZnO. At 240 °C, 6% SnO_2_-ZnO reaches a response of 140.27 to 10 ppm acetone, significantly higher than ZnO [83]. Response and recovery times are 108 s and 44 s, indicating a thicker electron depletion layer and better modulation during acetone adsorption. Sensitivity is linear in the 0.1–50 ppm range with R^2^ = 0.997 and a very low detection limit of 0.82 ppb, allowing accurate detection of breath acetone at clinically relevant levels such as 25.68 at 2 ppm. The improved properties result from n-n SnO_2_-ZnO heterojunctions, increased acetone adsorption on Sn sites (adsorption energy -1.149 eV), and a hollow mesoporous structure (BET 26.7 m^2^ g^−1^). Compared to leading acetone sensors, this system uniquely offers high responsiveness, an ultra-low detection limit, and stability, making it a strong candidate for real-world VOC monitoring. Interestingly, the Fe/Co MOF-hydrogel (Fe/Co MOF HG) sensor shows exceptional acetone gas sensing performance at room temperature [84]. It reaches a response of 12.3% at 500 ppb, roughly 2.4 times higher than the response of pristine Fe/Co MOF (5.1%). The hydrogel offers a broad linear detection range from 200 ppb to 4 ppm with a notable limit of detection (LOD) of 103 ppb, and it exhibits response and recovery times of 77 s and 75 s. Selectivity tests indicate a strong preference for acetone, with common interfering gases producing responses under 10%. Additionally, the hydrogel maintains 81.2% of its sensor response under 76% RH, compared to 71.3% for the pristine MOF, indicating good humidity resistance. Its mechanical flexibility is demonstrated by only a slight decrease in response from 12.3% to 11.6% after 230 bending cycles, and it shows consistent repeatability over multiple exposures. Structurally, the higher surface oxygen species (24.58%) and hydrogen bonds with acetone’s carbonyl groups improve sensitivity. Importantly, unlike many MOF-based sensors requiring 240–290 °C, the Fe/Co MOF HG can operate at room temperature with sub-ppm sensitivity, making it a promising option for wearable, flexible, and low-power sensors in healthcare and environmental monitoring. On the other hand, the UiO-66(Zr) preconcentrator paired with a ZrO_2_ cataluminescence (CTL) sensor shows outstanding gas detection capabilities for acetone [85]. It boosts the CTL signal roughly 25-fold, offering a linear detection range of 10–300 ppm with R^2^ = 0.9933 and a LOD of 2.79 ppm, suitable for diabetic breath analysis where acetone usually exceeds 1.8 ppm. Response and recovery occur within a few minutes, enabling near-real-time monitoring. The system exhibits strong selectivity, with only minor interference from ethanol and isopropanol, and humidity has little effect on CTL intensity even at 90% RH. Accuracy in mixed gases reaches about 91.3%, with a reproducibility of 6.8% RSD across parallel adsorption tubes and a short-term precision of 1.9% RSD. UiO-66 retains its structure and a high surface area (~1095 m^2^/g) after over 100 adsorption/desorption cycles, ensuring reusability and long-term stability. Optimized parameters such as detection at 253 °C, desorption at 314 °C, and a 10-min sampling balance sensitivity with durability. Overall, this sensor combines high sensitivity, stability, and humidity tolerance, making it a promising platform for portable acetone sensors, though further lowering of the LOD is needed to detect healthy breath levels. Fascinatingly, the Fe-based MOF VNU-15 demonstrates excellent low-temperature chemiresistive detection of acetone, with impressive behaviour [86]. At 50 °C, the sensor exhibits responses (R_a_/R_g_) of 1.30, 1.46, and 1.68 at 1, 5, and 10 ppm, indicating high sensitivity within clinically relevant concentration ranges. Selectivity tests show a strong preference for acetone, with R_acetone_/R_x_ ratios of 1.43 (CO), 1.52 (C_6_H_6_), and 1.55 (C_7_H_8_). The device responds in about 64 s and recovers in roughly 166 s at 10 ppm, maintaining consistent performance over several cycles with only moderate response reduction (from 1.683 to 1.513 over 15 days), demonstrating outstanding durability. Increased humidity up to 90% RH lowers the response due to competitive adsorption, while co-exposure to NO_2_ suppresses acetone signals, indicating oxidative interference. Mechanistically, O_2_^−^ ions dominate surface chemistry below 100 °C, where acetone reacts with ionosorbed oxygen, releasing electrons, supported by CO_2_ detection in gas analysis. Its high surface area (735 m^2^/g), Fe(II)/Fe(III) active sites, and proton-conductive channels work synergistically to enhance charge transfer. Unlike traditional MOF sensors operating at 150–450 °C, VNU-15 detects acetone selectively at 50 °C below the ~1.8 ppm threshold used for diagnosing diabetic ketosis, making it a promising candidate for portable, low-power breath sensors. Attractively, the UiO-66-NH_2_/chitosan (CS)/ionic liquid (IL) mixed-matrix membrane-based sensor shows promising gas-sensing abilities, especially for detecting acetone, which is important for non-invasive diabetes testing [87]. Operating best at 60 °C, the sensor detects as little as 1.06 ppm of acetone and responds strongly (~107% at 100 ppm) compared to the CS/IL base (~18.8%). This highlights the MOF filler’s role in boosting sensitivity. Response and recovery are quick 23 s and 18 s, which is faster than many metal oxide sensors that often need hundreds of seconds. Selectivity tests confirm acetone as the main target, due to strong hydrogen bonds between its carbonyl group and the -NH_2_ groups of UiO-66-NH_2_ and chitosan. Humidity reduces responses from about 85% in dry air to 54% at 50% RH and 13% at 90% RH, but performance remains effective under physiological breath moisture (50–74%). The sensor also shows excellent repeatability of nearly 85.4% and stability of 89.4% over multiple cycles. The sensing mechanism involves acetone adsorption and hydrogen bonding, which disrupt protonic and ionic pathways in the polymer-IL network. Overall, this sensor provides low-temperature, selective, and fast acetone detection, offering a competitive alternative to traditional high-temperature oxide sensors.

Recent research shows that these strategies significantly enhance ethanol sensing performance. Thin-film MOFs offer selective recognition at room temperature through coordinative binding, while MOF-derived oxides and composites add hierarchical porosity, heterojunctions, and catalytic interfaces. These features speed up response and recovery cycles and enable detection of trace concentrations. The next section presents key examples of these developments, illustrating the various structure–function relationships that drive their gas-sensing capabilities. Interestingly, the Zn_3_(btc)_2_.12H_2_O (Zn-btc) novel films studied here demonstrate effective room-temperature gas sensing for primary alcohols, monitored by shifts in the Kelvin probe work function (Φ) [88]. Structurally, this MOF features Zn^2+^ centers octahedrally coordinated to carboxylate oxygens and water ligands, forming hydrophilic sites amid hydrophobic btc phenyl linkers. Unlike in porous MOFs, adsorption occurs through ligand-exchange interactions, where alcohols reversibly replace coordinated water molecules. The hydroxyl group binds to Zn^2+^, while the alkyl chain interacts with btc linkers via van der Waals forces, altering electronic states and leading to measurable Φ shifts. The response strength correlates with alcohol chain length (propanol > ethanol > methanol) and vapor concentration, showing both sensitivity and selectivity. Humidity significantly impacts performance because water competes for Zn^2+^ binding, lowering the Φ response under high relative humidity compared to dry conditions. Notably, chain-length discrimination results from a combination of Lewis acid–base interactions at Zn^2+^ and dispersion forces with aromatic linkers. On the other hand, a novel Co_3_O_4_/Ti_3_C_2_T_x_ composite derived from MOF shows excellent ethanol sensing performance, particularly when Ti_3_C_2_T_x_ is loaded at 2 wt% [89]. The highest response (R_a_/R_g_) to 50 ppm ethanol reaches 190 at 200 °C, surpassing the performance of pure Co_3_O_4_. Dynamic testing shows quick response (~50 s) and recovery (~45 s) at this temperature, signifying an 18-fold enhancement compared to the unmodified oxide. The sensitivity increases with ethanol concentration and is still noticeable at 1 ppm, where pure Co_3_O_4_ is nearly unresponsive, highlighting MXene’s crucial role in enabling detection at low ppm levels. In addition, selectivity analyses confirm a strong inclination for ethanol over other interfering gases, while stability results show over 90% response retention after 60 days with consistent cyclic recovery. The difference in work function between Co_3_O_4_ (5.35 eV) and Ti_3_C_2_T_x_ (3.9 eV) forms a Schottky barrier, which enhances resistance modulation. Benchmarking shows superior performance compared to other Co_3_O_4_ hybrids, confirming the heterointerface design’s effectiveness for fast, selective, and stable ethanol detection. Furthermore, Ag-ZnO hollow nanocages derived from MOFs demonstrate exceptional ethanol gas sensing capabilities owing to their distinctive structure and catalytic interfaces [90]. These hollow, mesoporous Ag-ZnO nanocages with evenly distributed Ag nanoparticles offer numerous active sites and efficient gas diffusion channels. At 250 °C, the Ag-ZnO showed a response of approximately 84.6 to 100 ppm ethanol, significantly surpassing pure ZnO, with a quick response time of about 5 s and recovery in around 10 s. Notably, the detection limit was as low as 23.1 ppb, emphasizing its ultra-high sensitivity. Mechanistically, adding Ag increased the surface area, facilitated oxygen adsorption, and created Schottky junctions with ZnO. This raised the baseline resistance and improved modulation during ethanol detection. XPS analysis showed an increase in chemisorbed oxygen species, and UPS indicated a higher work function, suggesting stronger electronic interactions at the metal–semiconductor interface. Oxygen dependence experiments demonstrated that O^−^ species primarily drive the sensing process, with ethanol reacting with adsorbed oxygen to release electrons into ZnO. In addition, Fe-Co-Ni trimetallic oxides derived from Fe-MIL-88B show exceptional ethanol sensing capabilities due to their mesoporous structure and rich heterojunctions [91]. The optimized Fe_7_Co_1.5_Ni_1.5_ sample achieves a high response (~71.9 to 100 ppm ethanol) at 250 °C, with good selectivity, fast response (~35 s) and recovery (~26 s), and stable operation over 60 days. The sensing mechanism involves oxygen adsorption–desorption and redox reactions with ethanol, where electron release changes resistance. The presence of α-Fe_2_O_3_ (n-type) alongside CoFe_2_O_4_ and NiFe_2_O_4_ (p-type) forms numerous p-n junctions, boosting resistance variations and enabling composition-dependent polarity switching. Fe-rich materials act as strong n-type ethanol sensors, while Co/Ni-rich ones serve as p-type sensors with lower responses. Compared to other MOF-derived oxides, this trimetallic system excels due to synergistic catalysis, mesoporosity, and interface engineering. Although high operating temperatures remain a challenge, its polarity switching, low detection limit, and reproducibility make it a promising platform for ethanol sensing. Future efforts should aim to reduce operating temperature, enhance humidity resistance, and integrate these materials into practical devices for real-world use. Fascinatingly, the Au-decorated ZnO, made from ZIF-8 templates, exhibits improved ethanol sensing due to combined structural and electronic effects [92]. The mesoporous ZnO, with a high surface area and many oxygen vacancies, allows quick gas diffusion and dense active sites. Adding about 1.8 wt% Au forms Schottky junctions at the Au-ZnO interface, raising resistance and improving ethanol sensing response. Au also helps dissociate oxygen, increasing surface oxygen coverage and speeding up redox reactions with ethanol. As a result, the sensor reaches a high response (~37.7 at 100 ppm) at a relatively low temperature of 250 °C, with quick response and recovery times (around 19 s/9 s) and good repeatability. It can detect ethanol as low as 1 ppm with a linear response and maintains selectivity over methanol, acetone, and other interfering gases. DFT calculations confirm that ethanol adsorbs more strongly on Au/ZnO than on plain ZnO, supporting the chemical sensitization mechanism. However, challenges such as humidity effects and long-term durability need further study.

Furthermore, the gas-sensing ability of a Cu-BTC (MOF-199) thin-film chemicapacitor, which detects gases through changes in dielectric constant [93]. The porous MOF layer, with a surface area of approximately 705 m^2^g^−1^ and a pore size of about 0.53 nm, offers plentiful adsorption sites for vapors. Room temperature measurements revealed the most linear responses at 1 MHz, due to dielectric relaxation effects. Methanol showed stronger responses than ethanol, aligning with its higher polarity, with detection thresholds around 47 ppm for methanol and 150 ppm for ethanol. Stepwise exposure tests confirmed reversible adsorption–desorption cycles with consistent results over multiple runs. Significantly, the sensor design, featuring a porous Ag top electrode, promotes analyte diffusion into the MOFs, improving response reliability. The findings emphasize advantages like operation at room temperature, a clear polarity-based signal direction, and reliable short-term cycling. Limitations include moderate detection limits, frequency-dependent operation, absence of detailed response/recovery kinetics, and possible humidity effects. The Zn-based MOF [Zn_3_(cpoip)_2_(4,4’-bpy)_2_·H_2_O] demonstrates excellent luminescence-based sensing for methanol owing to its size-selective channels and Zn-O cluster-centered emissive states [94]. When dispersed in ethanol, the framework exhibits a response to methanol, with a new emission at ~404 nm, while ethanol itself shows negligible interaction. This discrimination arises from aperture-controlled guest selectivity: methanol (kinetic diameter ~3.8 Å) can diffuse into the ~4.6 Å channels, while ethanol (~5.1 Å) is sterically excluded. Such guest-induced modulation of Zn-O electronic states generates a strong optical readout, highlighting the role of structural confinement in achieving selective chemical sensing. The sensor achieves a remarkably low detection limit (~2 × 10^−7^ *v*/*v*, ~0.2 ppm) in ethanol, with a log-log linear calibration enabling straightforward quantification up to 0.01 *v*/*v*. Water competes for binding, raising the detection threshold in aqueous matrices, although spiked beverage tests confirm practical utility. Key strengths include the modality, intrinsic selectivity via channel size, and reproducible calibration. Nonetheless, this work provides a blueprint for gas-sensing MOFs, stabilize the framework, engineer hydrophobic channels, and exploit guest-induced luminescence for selective, sub-ppm methanol detection. In addition, a novel Fe-BTC (Basolite F300) has promising gas-sensing capabilities, especially for hydrophilic vapors like water, methanol, and ethanol [95]. Impedimetric tests indicate that Fe-BTC selectively reacts with polar analytes and exhibits minimal response to common interferents such as O_2_, CO_2_, C_3_H_8_, NO, and H_2_. Among all analytes tested, water vapor achieved the highest sensitivity, offering linear, reversible responses at 120 °C with minimal baseline drift, outperforming traditional polymer humidity sensors. Frequency-dependent analysis confirmed increased sensitivity at low frequencies, emphasizing charge transport dynamics as a key sensing mechanism. Humidity interference remains a significant issue, particularly for hydrophilic MOFs, where water molecules compete with alcohol at metal centers. Moreover, the high operating temperatures required for many MOF-derived oxides limit their application in portable and wearable sensors. Such advancements could enable practical alcohol sensors that leverage the MOF film’s capacity to operate at room temperature while maintaining the high sensitivity and rapid response of MOF-derived composites. Fascinatingly, the Eu-HODA metal–organic framework demonstrates unique dual-mode luminescent gas sensing properties [96]. When excited at 305 nm, the framework exhibits strong Eu^3+^ emissions, which respond distinctly to different volatile analytes. Methanol induces a significant effect, nearly tripling the emission intensity at 614 nm. This is attributed to hydrogen bonding with coordinated water molecules that reduces non-radiative quenching, while the pore size (~4.1 Å) perfectly accommodates methanol (3.8 Å), enabling efficient interactions. Larger alcohols such as ethanol and n-propanol show diminished or negligible responses, confirming a size-selective mechanism. This sensitivity surpasses previous MOF-based acetone sensors, making it highly practical for real-world monitoring. Importantly, Eu-HODA shows excellent stability, maintaining crystallinity after repeated sensing-regeneration cycles and extended solvent exposure.

Interestingly, the MOF-derived porous SnO_2_ submicron cubes exhibit promising gas-sensing performance toward n-butanol at room temperature [97]. The hollow cubic morphology and abundant oxygen vacancies provide high surface reactivity and efficient adsorption sites, enabling a strong response behavior. This is evident in the responses of 175% at 100 ppm and 250% at 500 ppm, with the response fitting well to a power law, allowing for calibration. Selectivity tests against common interfering vapors show a clear preference for n-butanol, demonstrating intrinsic specificity without the need for dopants or heterostructures. Response and recovery times (~184 s/183 s) are acceptable for room-temperature operation, but slower than those of conventional heated sensors. Humidity studies reveal reduced response at increasing RH up to 50%, after which performance stabilizes, suggesting partial water interference. Repeatability is high in short-term cycling, while long-term stability shows a ~31% drop over 16 days. Overall, the work highlights that MOF-derived porous SnO_2_ can achieve selective room-temperature VOC detection through defect engineering and pore design. On the other hand, calcination temperature greatly affects the gas sensing ability of MOF-derived Co_3_O_4_ nanospheres [98]. The Co_3_O_4_-400 had the highest response to n-butanol, with a sensitivity of 53.78 at 100 ppm and an optimal operating temperature of 140 °C. This improved performance is due to its porous structure, moderate surface area, and the highest oxygen vacancy content (53.7%), which offer plenty of active sites for oxygen adsorption and catalytic reactions. Dynamic tests of Co_3_O_4_-400 showed quick response and recovery times (99 s/50 s), outperforming other samples. A linear response was noted between 3–20 ppm, with a detection limit around 150 ppb, highlighting high sensitivity at low levels. Selectivity tests revealed a strong preference for n-butanol over other VOCs, with responses 1.3–5.5 times higher; xylene was the only compound causing noticeable interference. However, the p-type semiconducting nature of Co_3_O_4_ promotes hole buildup in air, and when n-butanol is reduced, it interacts with adsorbed O^−^ species, releasing electrons and raising resistance. The sensor demonstrated strong stability, retaining approximately 87% of its original response after 45 days. Overall, this work highlights the importance of oxygen-vacancy engineering and porous nanostructures in enabling selective VOC sensing at low temperatures. Remarkably, the Cr_2_O_3_/RGO heterojunction sensor shows outstanding gas-sensing performance for n-butanol, due to the combined effects of MOF-derived Cr_2_O_3_ nanostructures and conductive reduced graphene oxide [99]. The best-performing composite (RC-2, with about 2.9 wt% RGO) achieves a response of approximately 121–124 at 100 ppm and 160 °C, surpassing pure Cr_2_O_3_. Its response peaks at an optimal temperature, exhibiting a volcano-shaped dependence where surface reactions improve at moderate heat but desorption speeds up at higher temperatures. Response and recovery times are around 150 s and 250 s, respectively, moderate but consistent. Notably, the sensor maintains a linear response from 50 ppb to 100 ppm and has an exceptionally low detection limit of 8.6 ppb. Humidity, however, lowers sensitivity by reducing chemisorbed oxygen and blocking adsorption sites, which could potentially be mitigated by using hydrophobic coatings or adjusting the signal. The sensing mechanism mainly involves oxygen ion adsorption and consumption, affecting the hole accumulation layer in this p-type material. Excess RGO can impair performance due to shunting and limited diffusion. Furthermore, the quasi-Zn-MOF-derived ZnO sensors demonstrate outstanding gas-sensing properties toward n-butanol due to their defect-rich surfaces and partially preserved framework structure [100]. Annealing Zn-MOF at 160–210 °C generates ZnO nanocrystallites enriched with oxygen vacancies (O^−^_v_), which act as active sites for oxygen chemisorption and electron exchange. At the optimum operating temperature of 200 °C, Zn-MOF-160 achieves a high response (~204 at 30 ppm), while Zn-MOF-210 shows both excellent sensitivity (response ~538 at 100 ppm) and rapid response/recovery times (~3–6 s). The detection limit reaches ~0.43 ppm, highlighting strong low-concentration detection capability. Importantly, the superior sensing performance is linked not to surface area but to O^−^_v_ density and unsaturated Zn centers that enhance interaction with n-butanol molecules. Selectivity tests confirm a clear preference for n-butanol over interfering gases, attributed to stronger binding and faster electron return kinetics. However, humidity reduces performance by blocking active sites, and long-term stability is more reliable for quasi-MOFs annealed ≥210 °C compared to pristine MOFs. The mechanism follows the n-type metal oxide pathway, where adsorbed oxygen reacts with reducing gases to modulate the electron-depletion layer. On the other hand, the Tourmaline@ZnO core–shell nanostructures derived from ZIF-8 exhibit superior n-butanol sensing properties compared with pristine ZnO [101]. The optimized 5 wt% composite achieved a high response of 294.4 at 100 ppm and 320 °C, with faster response/recovery times and remarkable selectivity. This enhancement is attributed to several synergistic effects: (i) increased surface area and mesoporosity facilitating gas adsorption, (ii) higher surface oxygen-vacancy content (43.3% vs. 33.2% in ZnO) providing abundant active sites for O_2_ chemisorption, and (iii) suppressed electron–hole recombination and slight band-gap narrowing that improve charge transfer kinetics. Importantly, the spontaneous polarization of tourmaline modulates the ZnO band structure, deepening depletion or accelerating charge transport depending on field orientation, which strengthens both sensitivity and kinetics. The composite also demonstrated linear response–concentration relationships with a lower detection limit (0.037 ppm vs. 0.391 ppm) and reduced cross-sensitivity in mixed VOC environments. However, like most MOS sensors, humidity significantly suppressed response due to competitive adsorption, and operation required elevated temperatures (~320 °C). Stability tests confirmed minimal drift over 30 days, highlighting robustness. Furthermore, a Cu-carborane metal–organic framework (mCB-MOF-1) shows excellent gas-sensing properties due to its hydrophobic nature, stability in water, and ability to selectively adsorb vapors [102]. Its surface features large water contact angles and minimal water uptake, demonstrating strong humidity resistance, a vital trait for stable sensors in real-world settings. Even after long exposure to hot water and varying pH levels, mCB-MOF-1 stays crystalline and porous, ensuring long-term durability. Gas adsorption tests display steep type-I isotherms for alcohols, especially n-butanol, at low pressures, indicating robust interactions with the framework that enable high sensitivity at trace levels. Notably, the framework exhibits a high butanol/ethanol selectivity (>12 at low pressure), outperforming ZIF-8, which is valuable for detecting butanol amid common interferents. Computational studies show this is due to the interpenetrated pore structure, hydrophobic B-H/B-H/phenyl-rich surfaces, and stronger binding energies, all of which favor butanol adsorption. Dynamic gas chromatography confirms stronger butanol binding, though with slower diffusion, implying high sensitivity but longer response and recovery times, which can be addressed through thin-film coatings or mild heating. Additionally, Fe-doped MOF-derived NiO shows excellent n-butanol sensing due to combined structural and electronic effects [103]. The optimized composition (1.5 at%) displays a hierarchical nanosheet-particle structure, offering many active sites and aiding gas diffusion. At 275 °C, it achieves a high response (114 to 100 ppm n-butanol) with a very low detection limit of 50 ppb, outperforming undoped NiO by nearly two orders of magnitude. Response and recovery times (63 s/21 s) are also much faster, indicating improved surface reaction kinetics. Selectivity tests confirm a strong preference for n-butanol over common interfering gases, whereas higher Fe loadings reduce specificity. Mechanistically, Fe^3+^ doping enhances NiO by introducing catalytic centers, increasing Ni^3+^/Ni^2+^ ratios, and enriching oxygen vacancies, as confirmed by XPS. These modifications strengthen charge depletion layers and accelerate oxygen chemisorption–desorption dynamics. DFT further supports this, showing stronger n-butanol adsorption and larger charge transfer on Fe-doped surfaces, which explains the amplified resistance modulation. Despite excellent reproducibility and interference resistance, long-term stability remains a challenge, with partial decline after 30 days.

**Figure 7:**
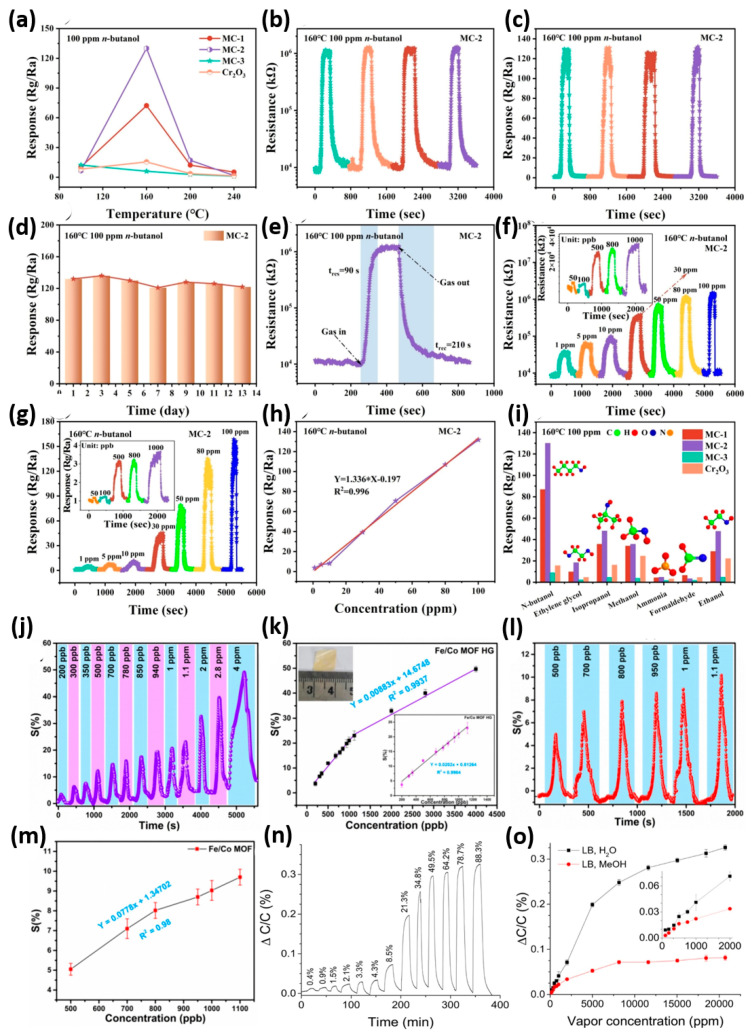
(**a**–**i**) Gas sensing performance of Cr_2_O_3_/MXene composites toward n-butanol detection. (**a**) Response of sensors (Cr_2_O_3_, MC-1, MC-2, MC-3) as a function of operating temperature toward 100 ppm n-butanol. (**b**) Dynamic response–recovery curves of MC-2 to 100 ppm n-butanol over four consecutive cycles at 160 °C. (**c**) Resistance stability of MC-2 during repeated n-butanol exposure. (**d**) Long-term stability of MC-2 over 13 days. (**e**) Response and recovery times of MC-2 at 160 °C. (**f**) Response curves of MC-2 to various n-butanol concentrations (50 ppb–100 ppm). (**g**) Calibration curve showing the linear response behavior of MC-2. (**h**) Corresponding root-mean-square (RMS) noise level for LOD estimation. (**i**) Selectivity of MC-2 toward 100 ppm of various volatile organic compounds (VOCs) at 160 °C, demonstrating preferential sensitivity to n-butanol. Reproduced with permission from Ref. [104]. Copyright (2024) Elsevier. (**j**–**m**) Dynamic response and linear regression plots of Fe/Co MOF hydrogel (HG) and Fe/Co MOF sensors toward varying concentrations of acetone, demonstrating superior sensitivity, wider dynamic range, and lower detection limit for the hydrogel sensor. (**j**) Dynamic response of the Fe/Co MOF hydrogel (HG) sensor to varying concentrations of acetone (200 ppb to 4 ppm) at room temperature, showing a consistent increase in sensing response. (**k**) Linear regression analysis of Fe/Co MOF HG sensor demonstrating excellent linearity and a low LOD of 103 ppb. (**l**) Dynamic response of the Fe/Co MOF sensor to acetone concentrations ranging from 500 ppb to 1.1 ppm, indicating a limited response range. (**m**) Linear regression curve for the Fe/Co MOF sensor, showing saturation behavior beyond 800 ppb and reduced sensitivity compared to the hydrogel sensor. Reproduced with permission from Ref. [84]. Copyright (2024) Elsevier. (n, o) illustrates the gas sensing performance of capacitive sensors made with MIL-96(Al) metal–organic framework (MOF). (**n**) Real-time capacitive response of MIL-96(Al) Langmuir–Blodgett (LB) film-based interdigitated electrode (IDE) sensors exposed to increasing relative humidity (RH) levels, demonstrating reversible and reproducible behavior across multiple humidity cycles. (**o**) Normalized capacitance change in the sensors as a function of methanol and water vapor concentration, showing significantly higher sensitivity to water at concentrations above 500 ppm. Reproduced with permission from Ref. [105]. Copyright (2020) American Chemical Society.

The gas sensing performance of Cr_2_O_3_/MXene composites prepared via the hydrothermal method is thoroughly examined in Figure 7a–i [104]. Among the samples tested, the MC-2 composite, with 6 wt% MXene, showed the best response to n-butanol gas. As shown in Figure 7a, the sensor response varies with temperature, initially increasing, then declining. This behavior results from the balance between enhanced surface chemical activity and the thermal desorption of gas molecules. The optimal temperature for MC-2 was identified as 160 °C, where the sensor achieved a remarkable response of 130.1 to 100 ppm of n-butanol. This response is 8.4 times greater than that of pure Cr_2_O_3_ (15.5), demonstrating the significant impact of MXene addition and heterojunction formation on sensor performance. The MC-2 sensor demonstrated excellent repeatability and stability over time. Figure 7b,c shows that its resistance and response values remained steady through four consecutive exposure cycles to 100 ppm n-butanol at 160 °C, indicating reliable performance. Additionally, as seen in Figure 7d, the sensor’s response was consistent over 13 days, confirming its robustness for long-term use. The response and recovery behaviors, detailed in Figure 7e, show times of 90 s and 210 s, respectively. These quick response times are due to MXene’s high conductivity, the electron-sensitizing C-dots, and the multiple heterojunctions within the composite, all of which promote fast charge transfer and gas interaction. A key feature of the MC-2 sensor is its high sensitivity and low detection limit. As shown in Figure 7f,g its response increases linearly with n-butanol concentrations from 50 ppb to 100 ppm. The response–concentration fit, Y = −0.197 + 1.336X, with an R^2^ of 0.996, highlights a strong linear relationship. Based on this slope and the RMS noise, the LOD was estimated at just 10.1 ppb. This remarkably low LOD positions the MC-2 sensor among the most sensitive n-butanol sensors available, making it suitable for detecting trace amounts of volatile organic compounds in the real world. The MC-2 sensor demonstrated outstanding selectivity for n-butanol compared to other volatile organic compounds (VOCs), as shown in Figure 7i. When exposed to 100 ppm of various VOCs, including ethanol, formaldehyde, methanol, isopropanol, ammonia, and ethylene glycol, the sensor consistently had the strongest response to n-butanol, with signals approximately 3 to 38 times higher than those for other gases. This high selectivity primarily results from specific surface interactions facilitated by the composite’s structure, where Cr_2_O_3_, TiO_2_ (derived from oxidized MXene), and C-dots collaborate to promote selective adsorption and redox reactions. On the other hand, Figure 7j–m shows the dynamic response and linear regression plots of Fe/Co MOF hydrogel (HG) and Fe/Co MOF sensors across different acetone concentrations, highlighting the hydrogel sensor’s superior sensitivity, wider dynamic range, and lower detection limit [84]. The gas sensing properties in Figure 7j show that the Fe/Co MOF hydrogel (HG) sensor outperforms others in detecting acetone at room temperature, offering high sensitivity and a broad dynamic range. When exposed to acetone concentrations from 200 ppb to 4 ppm, the sensor’s response increased steadily. At 200 ppb, the response was 3.7%, climbing sharply to 49.6% at 4 ppm. This notable response growth confirms the hydrogel’s effective interaction and adsorption of acetone molecules over a wide concentration range. The linear regression analysis, as shown in Figure 7k, further supports the sensor’s effectiveness by indicating excellent linearity in response across both low and high acetone concentrations. With correlation coefficients (R^2^) of 0.9904 and 0.9937, respectively, for different concentration ranges, the sensor maintains consistent and reliable readings. Additionally, the slope of the regression curve is 0.02 at lower concentrations and 0.0088 at higher concentrations, suggesting that while the sensor continues to respond to increasing acetone levels, a degree of surface saturation begins to influence the sensitivity at higher doses. Nevertheless, the sensor demonstrated an impressive LOD of just 103 ppb, making it highly suitable for applications requiring trace-level acetone detection. In contrast, the Fe/Co MOF-based sensor showed inferior performance. Figure 7l,m reveals that while the powder sensor exhibited some response to acetone, the sensitivity plateaued beyond 800 ppb. For instance, as the acetone concentration increased from 800 ppb to 1.1 ppm, the response rose marginally from 7.9% to 9.5%. This non-linear behavior indicates surface saturation, likely due to limited porosity and fewer active adsorption sites, which restrict the sensor’s dynamic performance. These limitations highlight the advantage of the hydrogel structure, which supports enhanced diffusion and adsorption of gas molecules. Therefore, it is concluded that the Fe/Co MOF HG sensor shows a dynamic range, low detection limit, high sensitivity, and excellent linearity, making it an excellent candidate for real-time, low-power acetone detection. Figure 7n,o highlights the gas sensing performance of capacitive sensors fabricated using MIL-96(Al) MOF nanoparticles deposited as Langmuir–Blodgett (LB) thin films on interdigitated electrodes (IDEs) [105]. Figure 7n illustrates the sensor’s dynamic capacitive response as relative humidity (RH) increases. The sensor shows step-wise rises in capacitance aligned with higher RH levels, featuring clear and sharp response and recovery phases. This indicates that the MIL-96(Al) LB sensor has excellent water vapor sensitivity and exhibits quick, reversible adsorption–desorption cycles. Additionally, the baseline capacitance resets to its initial value after each cycle, confirming the sensor’s high repeatability, an important feature for reusable gas sensors. Figure 7o further examines the sensor’s sensitivity differences to water and methanol vapors. At lower concentrations (below 500 ppm), both analytes trigger similar responses, indicating the sensor’s responsiveness within this range. However, above 500 ppm, water produces a notably stronger response, with capacitance changes up to four times greater than those for methanol at 5000 ppm. This suggests that the MOF thin film has a higher affinity for water molecules at elevated analyte levels. Therefore, it is concluded that the gas sensing properties in Figure 7n,o confirm that MIL-96(Al) LB films are highly responsive to water vapor and moderately sensitive to methanol. These characteristics demonstrate the potential of MIL-96(Al)-based capacitive sensors for practical applications in environmental monitoring and industrial safety, especially for distinguishing between humidity and alcohol-based vapors.

In the sensor platforms reviewed above, several patterns are evident. For improved selectivity at low temperatures, H_2_S sensors depend on bimetallic MOFs or formed oxides, such as CuO/ZnO. The detection of NO_2_ utilizes the redox activity and conductivity modulation of structures like MIL-47(V) and UiO-66 derivatives, which can achieve relatively low detection limits (<500 ppb). VOC sensors, particularly for acetone, utilize MOFs with tunable pore sizes and hybrid materials that enhance analyte diffusion and surface contact, with some recent sensors reaching sub-ppb sensitivity. Lastly, MOF-based humidity sensors or their oxide counterparts exhibit ultrafast response times (1 s) and low detection thresholds (<1% RH), primarily due to high porosity and polar surface chemistry. Furthermore, Table 1 presents a comparative overview of different gas response, response and recovery time, limit of detection, and stability based on sensors using MOFs or materials derived from MOFs. Therefore, it is concluded that these results confirm that optimizing MOF composition, morphology, and hybridization is one of the key factors in achieving high-performance sensing, which guides sensor development.

## 4. Conclusions and Future Perspectives

This review thoroughly investigates the strategic design and application of metal–organic frameworks (MOFs) for gas sensing. Key approaches, such as linker functionalization, metal node engineering, composite formation, and nanoscale structuring, have been demonstrated to significantly enhance gas sensor performance, including sensitivity, selectivity, response time, and stability.

Among the strategies derived from MOFs, heterostructure-based sensors like ZnO/CuO and CuO/In_2_O_3_ have shown superior H_2_S detection, leveraging p-n junction effects and oxygen vacancy engineering. Bimetallic structures, such as FeCu-MOFs, exhibit enhanced redox activity and improved conductivity, making them effective for room-temperature NO_2_ sensing. Mixed-matrix membranes, including MOFs-5 integrated into chitosan/glycerol and flexible films with sulfonated UIO-66-based polyelectrolytes, proved useful for humidity detection and wearable sensors. The review also emphasizes combining MOFs with 2D materials like MXenes, graphene, and conductive polymers, which achieve ultralow detection limits and enhanced signal transduction. The MOFs-based composites yield multifunctional sensors capable of simultaneously detecting NO_2_, humidity, and VOCs. Techniques such as defect engineering, surface functionalization, and the addition of catalytically active sites have been particularly effective in increasing selectivity for gases like formaldehyde and acetone, particularly in medical diagnostic applications.

Despite progress, challenges remain in sensor durability, cross-selectivity in mixed environments, and the scalability of MOF-based fabrication. Future research should focus on: (i) hydrophobic modifications and robust metal node incorporation for better moisture and temperature stability, (ii) defect-tuned and surface-engineered MOFs for gas discrimination in complex matrices, and (iii) integrating with microelectronic platforms (MEMS/NEMS) for real-time, low-power sensing. Combining materials chemistry with device engineering is vital to transforming lab-scale MOF sensors into practical devices for urban air quality, industrial safety, and healthcare diagnostics. Progress in scalable synthesis methods like inkjet printing and layer-by-layer assembly will be key for commercial use. Future MOF-based smart sensor networks could be significant for industry safety and urban air quality monitoring. Commercial success relies on scalable, cost-effective, and reproducible manufacturing techniques, including green, continuous processes, printable inks, and scalable deposition methods supporting mass production without sacrificing performance. Addressing these challenges will allow MOF-based gas sensors to become versatile, energy-efficient, and intelligent, with applications in industrial process protection, health improvement, precision medicine, and smarter cities. Interdisciplinary efforts are essential to harness MOFs’ unique properties for creating effective sensors.

## Figures and Tables

**Table 1:** Comparative analysis of various gases using MOFs or materials derived from MOFs.

S. No.	Sensor Materials	Test Gas	O.T.	R/LOD	t_response_	t_recovery_	Stability	Ref.
1	Cu-MOFs	H_2_S	R.T.	24 mV (100 ppb)/5 ppb	63 s	176 s	66 cycles	[106]
2	MOF-5/CS/IL	H_2_S	R.T.	91% (100 ppm)/—	8 s	30 s	97%	[61]
3	ZM600	H_2_S	200 °C	10% (1 ppm)/56.9 ppb	18 s	29 s	120 days	[107]
4	Uniform Cu-doped Zn_2_SnO_4_	H_2_S	275 °C	8 (10 ppm)/10 ppb	21 s	27 s	—	[108]
5	MOF-303-IL-SO_3_Na (50%)	Humidity	R.T.	3075	14.8 s	21.3 s	12 days	[109]
6	SnO_2_-M-OV-300	NO_2_	120 °C	11,677 (1 ppm)/0.001 ppm	33 s	15 s	30 days	[76]
7	Au_3_@WS_2_-2/Ni_3_(HITP)_2_	NO_2_	R.T.	14.73% (5 ppm)/0.5 ppm	97 s	110 s	55 days	[78]
8	Sn/Zn-ZIF-8	Acetone	240 °C	140.27 (10 ppm)/0.82 ppb	108 s	44 s	20 days	[83]
9	Fe/Co MOF HG with Cu metal	Acetone	R.T.	12.28 (500 ppb)/103 ppb	77 s	75 s	30 days	[84]
10	VNU-15	Acetone	50 °C	1.30 (1 ppm)/—	64 s	166 s	—	[86]
11	Metal–organic frameworks-derived Co_3_O_4_/Ti_3_C_2_T_x_ Mxene	Ethanol	200 °C	190 (50 ppm)/—	50 s	45 s	60 days	[89]
12	Metal–organic framework-derived ZnO hollow nanocages functionalized with nanoscale Ag catalysts	Ethanol	250 °C	84.6 (100 ppm)/23.1 ppb	5 s	10 s	—	[90]
13	FCN-MOSs	Ethanol	250 °C	71.9 (100 ppm)/30.7 ppb	35 s	26 s	60 days	[91]
14	Sn-MOF	n-butanol	R.T.	175% (100 ppm)/—	184 s	183 s	30 days	[97]
15	MOFs-derived Cr_2_O_3_/RGO p-p heterojunctions	n-butanol	160 °C	121.2 (100 ppm)/8.6 ppb	150 s	250 s	—	[99]
16	Zn-MOF-210	n-butanol	200 °C	173 (30 ppm)/—	3 s	1349 s	15 days	[100]
17	MOF-Derived tourmaline@ZnO nanostructure	n-butanol	320 °C	294.4 (100 ppm)/—	160 s	60 s	30 days	[101]
18	Iron-doped metal–organic framework-derived nickel oxide	n-butanol	275 °C	114 (100 ppm)/0.05 ppm	63 s	21 s	30 days	[103]
19	Metal–organic frameworks-derived Cr_2_O_3_/MXene composites	n-butanol	160 °C	130.1 (100 ppm)/10.1 ppb	90 s	210 s	13 days	[104]

O.T.—Operating temperature; R—Response; LOD—Limit of detection; t_response_—Response time; t_recovery_—Recovery time; R.T.—Room temperature; Ref.—References.

## Data Availability

Data are contained within the article.
